# Critical checklist of the Chalcidoidea and Mymarommatoidea (Insecta, Hymenoptera) of Germany

**DOI:** 10.3897/BDJ.10.e85582

**Published:** 2022-10-26

**Authors:** Stefan Vidal, Jochen Müller, Stefan Schmidt

**Affiliations:** 1 University of Göttingen, Göttingen, Germany University of Göttingen Göttingen Germany; 2 Friedrich-Schiller-University Jena, Jena, Germany Friedrich-Schiller-University Jena Jena Germany; 3 SNSB-Zoologische Staatssammlung München, Munich, Germany SNSB-Zoologische Staatssammlung München Munich Germany

**Keywords:** parasitoids, chalcidoid wasps, fauna, new records

## Abstract

**Background:**

Since the first checklist of German Chalcidoidea and Mymarommatoidea was published over two decades ago, a revision of the status of these superfamilies in Germany is overdue. The previous list contained chalcidoid species mentioned in published papers up to 2000 and was cross-checked with the data compiled by Noyes in the Universal Chalcidoidea Database. Additional species, determined by the first author, were also included. Since then, revisions of several chalcidoid genera have been published synonymising species or describing new species. The previous checklist also contained several erroneous names and doubtful records that turned out to be incorrect placements or questionable citations and are corrected in the present version.

**New information:**

The updated critical checklist of German Chalcidoidea includes 1,610 species from 19 families.

## Introduction

A revised and critical annotated checklist of the German Chalcidoidea and Mymarommatoidea updates the German checklist of Chalcidoidea published in 2001. The new checklist now includes 1,610 species from 19 families. The compiled list includes papers published since 2001 and several species names have been updated, based on the revisions of various genera published in the meantime.

The previous list included names of species described by Dalla Torre (1898), Förster (1841), Förster (1859), Förster (1860) and Förster (1861), or Ratzeburg (1844a), Ratzeburg (1844b), Ratzeburg (1848) and Ratzeburg (1852), many with a doubtful species status. Many of the species described by Förster are probably only colour variants, as for example, Schnee (2008) stated in his revision of the subfamily Anomaloninae (Hymenoptera: Ichneumonidae), based on historic type material from Förster that still exists.

Given the very well-recorded British insect fauna, Noyes calculated the proportion of Chalcidoidea species being about 8% (Noyes 2000). In Germany, around 33,000 insect species are known (Völkl and Blick 2004). Taking the same proportion of Chalcidoidea and Mymarommatoidea, it can be expected that at least 2,600 species of these two superfamilies are present in Germany. Species names follow Noyes (2019), with the database last updated in 2019. Several recent papers dealing with revisions of Eulophidae or Mymaridae (e.g. Hansson and Schmidt 2020, Triapitsyn 2021) have been taken into account.

## Materials and methods

The annotated checklist is based on the three main data sources: 1) The Universal Chalcidoidea Database, hosted by the Natural History Museum London (Noyes 2019), 2) the Fauna Europaea, hosted by the Museum for Naturkunde Berlin (de Jong et al. 2014; accessed December 2020) and 3) the SNSB-Zoologische Staatssammlung München (Schmidt 2021, accessed December 2020). Moreover, the first two authors of this paper have collected and determined several additional species, not yet included in these databases cited above. These specimens are in the private collections of these authors.

References are given for new additions to the checklist, i.e. species that were recorded since the last checklist (Vidal 2001) was published. References for older records are given, if not included in Noyes (2019).

## Data resources

### Checklist of German Chalcidoidea (Insecta, Hymenoptera)

Out of the 24 extant families of Chalcidoidea and Mymarommatoidea worldwide, 19 families are currently recorded from Germany (Table 1). The number of species listed by Mitroiu et al. (2015) exceeds 5,100 species for Europe. The recently-published checklist of British and Irish Chalcidoidea and Mymarommatoidea species comprises 1,754 species (Dale-Skey et al. 2016). Gijswijt compiled the Chalcidoidea species for the Netherlands and listed 1,133 species (Ulenberg 2016). The checklist by Vidal (2001) listed 1,778. Weber et al. (2018) list 1,939 species from Germany, based on the Universal Chalcidoidea Database (Noyes 2019), after removing synonyms, *nomina nuda* and fossil taxa (29 species) and adding four new records for Germany, but including species with doubtful species status.

The present critical checklist for Germany lists 1,610 species (Table 1, Table 2). The list excludes species with doubtful status or species that were not correctly cited in Vidal (2001) (see Table 3 below). The previous checklist by Vidal (2001), now corrected for species with doubtful status, comprises 1,328 species. The updated checklist thus adds 288 species as new for the German fauna.

### List of species excluded from the checklist of German species of Chalcidoidea

The species listed in Table 3 were excluded from the updated critical checklist of species recorded from Germany. We followed the same rationale as Horstmann (2001) for the checklist of German Ichneumonidae (see also Riedel et al. 2021). Horstmann (2001) included only species that were either described as new after 1945 or, for species described before that year, if the species were mentioned at least once in the entomological literature after 1945. Horstmann (2001) excluded species if they were mentioned only once in the literature before 1945 (i.e. in the original citation), because all of these species most likely are representing synonyms.

For the present checklist of German Chalcidoidea, we followed this principle in that we excluded species that were described by early authors during the 19^th^ century, including Dalla Torre (1898), Förster (1841), Förster (1859), Förster (1860), Förster (1861) and Ratzeburg (1844a), Ratzeburg (1844b), Ratzeburg (1848), Ratzeburg (1852) and that were not mentioned again in the literature after the original description. Since the type material is often regarded as being lost, an exact verification of the species status with previously or later described species is no longer possible (Bouček 1964, Graham 1969). Therefore, many of these species are expected to represent synonyms of known valid species from Germany. By excluding these species from the checklist, an attempt was made to minimise the risk of including species with doubtful status. The species that were not included in the main checklist are listed in Table 3, with notes explaining the reason for excluding the species from the checklist.

## Figures and Tables

**Table 1.** Number of species of German Chalcidoidea by family in Vidal (2001) and in the present checklist. ^1^in Vidal (2001) under Aphelinidae; ^2^now placed in Eulophidae; ^3^in Vidal (2001) under Torymidae.

**Family**	**Vidal (2001)**	**Present checklist**
Aphelinidae	49	47
Azotidae	n/a	3^1^
Chalcididae	23	22
Elasmidae	6^2^	n/a
Encyrtidae	165	193
Eucharitidae	2	1
Eulophidae	443	442
Eupelmidae	32	42
Eurytomidae	111	101
Leucospidae	3	3
Megastigmidae	n/a	12^3^
Mymaridae	104	88
Mymarommatidae	1	1
Ormyridae	12	9
Perilampidae	40	19
Pteromalidae	663	497
Signiphoridae	2	2
Tetracampidae	11	9
Torymidae	93	93
Trichogrammatidae	18	26
**Total**	**1,778**	**1,610**

**Table 2.** Checklist of German Chalcidoidea and Mymarommatoidea. "CL" in the remarks column refers to species in the previous checklist (Vidal 2001).

**Family**	**Species**	**Remarks**	**Source of information**
**Aphelinidae**			
	*Aphelinusabdominalis* (Dalman, 1820)		Höller (1988)
	*Aphelinusannulipes* (Walker, 1851)		
	*Aphelinusargiope* Walker, 1839		
	*Aphelinusasychis* Walker, 1839		
	*Aphelinuschaonia* Walker, 1839		Anton et al. (2020)
	*Aphelinusdaucicola* Kurdjumov, 1913		
	*Aphelinusfusciscapus* (Förster, 1841)		
	*Aphelinushumilis* Mercet, 1927		Anton et al. (2020)
	*Aphelinuslongipennis* (Förster, 1841)		
	*Aphelinusmali* (Haldeman, 1851)		
	*Aphelinussemiflavus* Howard, 1908		
	*Aphelinusthomsoni* Graham, 1976		
	*Aphelinusvaripes* (Förster, 1841)		Höller (1988)
	*Aphytischilensis* Howard, 1900		
	*Aphytischrysomphali* (Mercet, 1912)		Hoffmann (2002)
	*Aphytisluteus* (Ratzeburg, 1852)		
	*Aphytismytilaspidis* (LeBaron, 1870)		
	*Aphytisproclia* (Walker, 1839)		
	*Centrodoraacridiphagus* (Otten, 1941)		
	*Centrodoraamoena* Förster, 1878		
	*Centrodoralocustarum* (Giraud, 1863)		
	*Centrodoratibialis* (Nees, 1834)		
	*Coccobiusannulicornis* Ratzeburg, 1852	CL01: *Coccobiustestaceus* Masi	
	*Coccophagusinsidiator* (Dalman, 1825)		
	*Coccophaguslycimnia* (Walker, 1839)		Hoffmann (2002)
	*Coccophaguspalaeolecanii* Yanosh, 1957		Hoffmann (2002)
	*Coccophaguspulchellus* (Förster, 1841)	CL01: *Coccobiusfoersteri* Dalla Torre	
	*Coccophagusscutatus* Howard, 1895		
	*Coccophagusscutellaris* (Dalman, 1825)		
	*Coccophagussemicircularis* (Förster, 1841)		Hoffmann (2002)
	*Diaspiniphagusmoeris* (Walker, 1839)	CL01: *Coccophagoidesmoeris* Walker	
	*Encarsiaaleurochitonis* (Mercet, 1931)	CL01: *Encarsiaauleurochitonis*, misspelling	Baermann, pers. comm.
	*Encarsiaaurantii* (Howard, 1894)		
	*Encarsiaberlesei* (Howard, 1906)		Hoffmann (2002)
	*Encarsiacitrina* (Craw, 1891)		Hoffmann (2002)
	*Encarsiafasciata* (Malenotti, 1917)		
	*Encarsiainaron* (Walker, 1839)		
	*Encarsiaintermedia* (Ferrière, 1961)		
	*Encarsialahorensis* (Howard, 1911)		
	*Encarsialeucaspidis* (Mercet, 1932)		Schmidt, pers. comm.
	*Encarsiamargaritiventris* (Mercet, 1931)		Baermann, pers. comm.
	*Encarsiaperniciosi* (Tower, 1913)		
	*Encarsiatricolor* Förster, 1879		
	*Eretmocerusmundus* Mercet, 1931		
	*Mariettapicta* (André, 1878)		
	*Pteroptrixbicolor* (Howard, 1898)		
	*Pteroptrixlongiclava* (Girault, 1915)		
**Azotidae**			
	*Ablerusatomon* (Walker, 1847)		Hoffmann (2002)
	*Ableruscelsus* (Walker, 1839)		
	*Ableruspinifoliae* (Mercet, 1912)		
**Chalcididae**			
	*Belaspidianigra* (Siebold, 1856)		
	*Brachymeriafemorata* (Panzer, 1801)		
	*Brachymeriaminuta* (Linnaeus, 1767)		von Heyden (1887)
	*Brachymeriamoerens* (Ruschka, 1922)		
	*Brachymeriaobtusata* (Förster, 1859)	CL01: *Brachymeriawalkeri* Dalla Torre	Schmidt, pers. comm.
	*Brachymeriaparvula* (Walker, 1834)	CL01: *Chalcisfemorata* Nees	
	*Brachymeriapodagrica* (Fabricius, 1787)		
	*Brachymeriarugulosa* (Förster, 1859)		
	*Brachymeriasecundaria* (Ruschka, 1922)		
	*Brachymeriatibialis* (Walker, 1834)		von Heyden (1887)
	*Brachymeriavitripennis* (Förster, 1859)		
	*Chalcisbiguttata* Spinola, 1808		
	*Chalcismyrifex* (Sulzer, 1776)		
	*Chalcissispes* (Linnaeus, 1761)		Hörren and Enss (2017)
	*Conuraxanthostigma* (Dalman, 1820)		
	*Euchalcismagna* (Bouček, 1952)	CL01: *Hockeriamagna* Bouček	
	*Haltichellarufipes* (Olivier, 1791)		von Heyden (1887), Anton et al. (2020)
	*Hockeriaunicolor* Walker, 1834		
	*Hybothoraxgraffii* Ratzeburg, 1844	CL01: *Hybpthoraxgraffi*, misspelling	
	*Neohybothoraxhetera* (Walker, 1834)		
	*Psilochalcisbenoisti* (Steffan, 1948)		
	*Psilochalcissubarmata* (Förster, 1859)		
**Encyrtidae**			
	*Acerophagusaustriacus* (Mercet, 1925)	CL01: *Pseudaphycusaustriacus* Mercet	
	*Acerophagusmalinus* (Gahan, 1946)	CL01: *Pseudaphycusmalinus* Gahan	Hoffmann (2002)
	*Adelencyrtusaulacaspidis* (Brèthes, 1914)		
	*Adelencyrtusintersectus* (Fonscolombe, 1832)	CL01: *Epitetracnemusintersectus* Fonscolombe	
	*Ageniaspisatricollis* (Dalman, 1820)		
	*Ageniaspisfuscicollis* (Dalman, 1820)		
	*Ageniaspistestaceipes* (Ratzeburg, 1848)	CL01: *Holcothoraxtestaceipes* Ratzeburg	
	*Aglyptusrufus* (Dalman, 1820)		
	*Anagyriettapantherina* Ferrière, 1955		
	*Anagyrusbelibus* (Walker, 1837)		
	*Anagyrusmatritensis* (Mercet, 1921)		
	*Anagyrusorbitalis* (Ruschka, 1923)		
	*Anagyrusschmuttereri* Ferrière, 1955		
	*Anagyrusschoenherri* (Westwood, 1838)		Hoffmann (2002)
	*Anagyrussecuricornis* Domenichini, 1953		
	*Anomalicorniatenuicornis* Mercet, 1921		Hoffmann (2002)
	*Anthemuspini* Ferrière, 1927		
	*Anusianasicornis* Förster, 1860		Hoffmann (2002)
	*Aphycoidesclavellatus* (Dalman, 1820)		Hoffmann (2002)
	*Aphycusapicalis* (Dalman, 1820)		
	*Aphycushederaceus* (Westwood, 1837)		
	*Aphycussumavicus* Hoffer, 1954		
	*Baeocharispascuorum* Mayr, 1876		Hoffmann (2002)
	*Blastothrixbrittanica* Girault, 1917		
	*Blastothrixerythrostetha* (Walker, 1847)		
	*Blastothrixhedqvisti* Sugonajaev, 1984		
	*Blastothrixhungarica* Erdös, 1959		Hoffmann (2002)
	*Blastothrixilicicola* Mercet, 1921		
	*Blastothrixklapaleki* Hoffer, 1963		
	*Blastothrixlongipennis* Howard, 1881		Hoffmann (2002)
	*Blastothrixmatesovae* Sugonjaev, 1964		
	*Blastothrixnikolskajae* Sugonjaev, 1965		
	*Blastothrixsericea* (Dalman, 1820)		Hoffmann (2002)
	*Blastothrixtruncatipennis* (Ferrière, 1955)		
	*Bothriothoraxaltensteinii* Ratzeburg, 1844		
	*Bothriothoraxclavicornis* (Dalman, 1820)		
	*Bothriothoraxintermedius* Claridge, 1964		
	*Bothriothoraxparadoxus* (Dalman, 1820)		
	*Bothriothoraxserratellus* (Dalman, 1820)		
	*Bothriothoraxwichmani* Ferrière, 1956		
	*Boucekielladepressa* Hoffer, 1954		
	*Cerapterocerusceladus* (Walker, 1838)		
	*Cerapterocerusmirabilis* Westwood, 1833		Hoffmann (2002)
	*Cerchysiussubplanus* (Dalman, 1820)		Hoffmann (2002), Anton et al. (2020)
	*Cercobelusjugaeus* (Walker, 1837)		
	*Charitopusfulviventris* Förster, 1860		
	*Cheiloneurusclaviger* Thomson, 1876		Hoffmann (2002)
	*Cheiloneuruselegans* (Dalman, 1820)		Anton et al. (2020)
	*Cheiloneurusglaphyra* (Walker, 1837)		
	*Cheiloneurusparalia* (Walker, 1837)		
	*Choreiainepta* (Dalman, 1820)		Anton et al. (2020)
	*Coelopencyrtusarenarius* (Erdös, 1957)		
	*Copidosomaagrotis* (Fonscolombe, 1832)		
	*Copidosomaaithyia* (Walker, 1837)		
	*Copidosomaalbipes* (Westwood, 1837)		
	*Copidosomaaretas* (Walker, 1838)		
	*Copidosomaboucheanum* Ratzeburg, 1844	CL01: *Copidosomahilare* (Ratzeburg)	
	*Copidosomacervius* (Walker, 1846)		
	*Copidosomachalconotum* (Dalman, 1820)		
	*Copidosomacuproviride* Springate & Noyes, 1990		
	*Copidosomadius* (Walker, 1837)		
	*Copidosomafilicorne* (Dalman, 1820)	CL01: *Copidosomageniculatum* (Dalman)	
	*Copidosomaflagellare* (Dalman, 1820)		
	*Copidosomafloridanum* (Ashmead, 1900)		
	*Copidosomafuscisquama* (Thomson, 1876)		
	*Copidosomagenale* (Thomson, 1876)		
	*Copidosomairacundum* Erdös, 1957		
	*Copidosomapeticus* (Walker, 1846)		
	*Copidosomaserricorne* (Dalman, 1820)		
	*Copidosomasosares* (Walker, 1837)		
	*Copidosomaterebrator* Mayr, 1876		
	*Copidosomathebe* (Walker, 1838)		
	*Copidosomatruncatellum* (Dalman, 1820)		
	*Copidosomavaricorne* (Nees, 1834)	CL01: *Paralitomastixvaricorne* Nees	
	*Dinocarsishemiptera* (Dalman, 1820)		
	*Dinocarsishofferi* Graham, 1966		
	*Discodesaeneus* (Dalman, 1820)		
	*Discodescoccophagus* (Ratzeburg, 1848)		Hoffmann (2002)
	*Discodesdifferens* Yasnosh, 1972		Hoffmann (2002)
	*Discodesencopiformis* (Walker, 1847)		
	*Echthroplexispuncticollis* (Thomson, 1876)		von der Dunk (2012)
	*Ectromafulvescens* Westwood, 1833		
	*Ectromareinhardi* (Mayr, 1876)		
	*Encyrtusalbitarsis* Zetterstedt, 1838		
	*Encyrtusaurantii* (Geoffroy, 1785)		
	*Encyrtusinfelix* (Embleton, 1902)		
	*Encyrtusinfidus* (Rossius, 1790)		Hoffmann (2002)
	*Encyrtusswederi* Dalman, 1820		Hoffmann (2002), Anton et al. (2020)
	*Ericydnusapterogenes* Mayr, 1876		
	*Ericydnusbaleus* (Walker, 1838)		
	*Ericydnuslongicornis* (Dalman, 1820)		
	*Ericydnusrobustior* Mercet, 1921		Hoffmann (2002)
	*Ericydnussipylus* (Walker, 1837)		Hoffmann (2002)
	*Ericydnusstrigosus* (Nees, 1834)		
	*Ericydnustheron* Trjapitzin, 1982		Hoffmann (2002)
	*Ericydnusventralis* (Dalman, 1820)		
	*Eucoccidophagussemiluniger* (Hoffer, 1959)		Hoffmann (2002)
	*Eugahaniafumipennis* (Ratzeburg, 1852)		
	*Eupoecilopodaperpunctata* (Masi, 1942)		
	*Eusemioncornigerum* (Walker, 1838)		
	*Eusemionlongipennis* (Ashmead, 1888)		
	*Habrolepisdalmanni* (Westwood, 1837)		Hoffmann (2002)
	*Helegonatopusdimorphus* (Hoffer, 1954)		
	*Heterococcidoxenusschlechtendali* (Mayr, 1876)		
	*Homalotylusephippium* (Ruschka, 1923)		
	*Homalotyluseytelweinii* (Ratzeburg, 1844)		
	*Homalotylusflaminius* (Dalman, 1820)		
	*Homalotylushemipterinus* (De, Stefani, 1898)		
	*Homalotylusplatynaspidis* Hoffer, 1963		
	*Isodromusvinulus* (Dalman, 1820)		
	*Ixodiphagushookeri* (Howard, 1908)		
	*Lamennaisiaambigua* (Nees, 1834)		
	*Lamennaisianobilis* (Nees, 1834)	CL01: *Negeniaspidiusnobilis* Nees	
	*Leptomastideabifasciata* (Mayr, 1876)		
	*Leptomastixepona* (Walker, 1844)		
	*Mahencyrtuscomara* (Walker, 1837)		
	*Mayrencyrtusimandes* (Walker, 1837)		
	*Mayridiamerceti* Trjapitzin, 1969		
	*Mayridiamyrlea* (Walker, 1838)		Hoffmann (2002)
	*Mayridiaprocera* (Mercet, 1921)		
	*Metaphycusasterolecanii* (Mercet, 1923)		
	*Metaphycuschermis* (Fonscolombe, 1832)		
	*Metaphycusdelos* Guerrieri & Noyes, 2000		Hoffmann (2002)
	*Metaphycusinsidiosus* (Mercet, 1921)		Hoffmann (2002)
	*Metaphycusmaculipennis* (Timberlake, 1916)		Hoffmann (2002)
	*Metaphycusmelanostomatus* (Timberlake, 1916)		Hoffmann (2002)
	*Metaphycusnitens* (Kurdjumov, 1912)		Hoffmann (2002)
	*Metaphycuspunctipes* (Dalman, 1820)		
	*Metaphycussilvestrii* Sugonjaev, 1970		Hoffmann (2002)
	*Metaphycusstagnarum* Hoffer, 1954		
	*Metaphycusunicolor* Hoffer, 1954		
	*Metaphycuszebratus* (Mercet, 1917)	CL01: *Metaphycusparvus* Mercet	Hoffmann (2002)
	*Microterysaeneiventris* (Walker, 1837)		
	*Microterysapicipennis* Bakkendorf, 1965		Hoffmann (2002)
	*Microteryschalcostomus* (Dalman, 1820)		
	*Microterysduplicatus* (Nees, 1834)		
	*Microterysferrugineus* (Nees, 1834)		
	*Microterysfuscipennis* (Dalman, 1820)		
	*Microteryshortulanus* Erdös, 1956		Hoffmann (2002)
	*Microteryslunatus* (Dalman, 1820)		Hoffmann (2002)
	*Microterysmasii* Silvestri, 1919		Hoffmann (2002)
	*Microteryssylvius* (Dalman, 1820)		Hoffmann (2002)
	*Microterystessellatus* (Dalman, 1820)	CL01: *Microterystesselatus*, misspelling	
	*Microterystrjapitzini* Yanosh, 1969		Hoffmann (2002)
	*Microteryszarina* (Walker, 1837)	CL01: *Aschitusarina* Walker	
	*Miramucora* Schellenberg, 1803		
	*Oobiuszahaikevitshi* Trjapitzin, 1963		
	*Ooencyrtusfulvipes* Hoffer, 1963		
	*Ooencyrtuskuvanae* (Howard, 1910)		
	*Ooencyrtustardus* (Ratzeburg, 1844)		
	*Ooencyrtustelenomicida* (Vassiliev, 1904)		
	*Ooencyrtusvinulae* (Masi, 1909)		
	*Parablastothrixmetatibialis* Erdös, 1955		
	*Parablastothrixmontana* Erdös, 1955		
	*Parablastothrixplugarui* Trjapitzin, 1971		
	*Parablatticidabrevicornis* (Dalman, 1820)		
	*Platencyrtusparkeri* Ferrière, 1956		
	*Prionomastixmorio* (Dalman, 1820)		
	*Prionomitusmitratus* (Dalman, 1820)		
	*Prionomitustiliaris* (Dalman, 1820)		
	*Pseudencyrtusidmon* (Walker, 1848)		
	*Pseudencyrtusmisellus* (Dalman, 1820)		
	*Pseudencyrtussalicisstrobili* (Linnaeus, 1758)		
	*Pseudleptomastixbrevipennis* (Ferrière, 1957)		Bachmeier (1965)
	*Pseudorhopustestaceus* (Ratzeburg, 1848)		Hoffmann (2002)
	*Psilophrystenuicornis* Graham, 1969		
	*Rhopusparvulus* (Mercet, 1921)		
	*Sectiliclavacleone* (Walker, 1844)		
	*Subprionomitusfestucae* (Mayr, 1876)		
	*Syrphophagusaeruginosus* (Dalman, 1820)		
	*Syrphophagusaphidivorus* (Mayr, 1876)		Höller (1988)
	*Syrphophagusherbidus* (Dalman, 1820)		
	*Syrphophagusmamitus* (Walker, 1837)		
	*Syrphophaguspertiades* (Walker, 1837)		
	*Syrphophagustaeniatus* (Förster, 1861)		
	*Tachinaephaguszealandicus* Ashmead, 1904		
	*Tetracnemoideapiceae* (Erdös, 1946)		
	*Tetracnemoideaspilococci* (Ferrière, 1957)		Bachmeier (1965)
	*Tetracnemusdiversicornis* Westwood, 1837		
	*Tetracnemusheydeni* (Mayr, 1876)		
	*Thomsoniscaamathus* (Walker, 1838)		
	*Trechnitesfuscitarsis* (Thomson, 1876)		
	*Trechnitesinsidiosus* (Crawford, 1910)	CL01: *Trechnitespsyllae* (Ruschka)	
	*Trichomasthusalbimanus* Thomson, 1876		
	*Trichomasthuscyaneus* (Dalman, 1820)		Hoffmann (2002)
	*Trichomasthuscyanifrons* (Dalman, 1820)		
	*Trichomasthusdignus* Khlopunov, 1987		
	*Trichomasthusfrontalis* Alam, 1957		Hoffmann (2002)
	*Tyndarichusmelanacis* (Dalman, 1820)		
	*Tyndarichusnavae* Howard, 1910		
	*Tyndarichusscaurus* (Walker, 1837)		
	*Zaommaeriococci* (Ferrière, 1955)		Hoffmann (2002)
	*Zaommalambinus* (Walker, 1838)		Hoffmann (2002)
**Eucharitidae**			
	*Eucharisadscendens* (Fabricius, 1787)		Bürgis (2010), Creutzburg and Müller (2019)
**Eulophidae**			
	*Aceratoneuromyiaclaridgei* Graham, 1991		
	*Aceratoneuromyiagranularis* Domenichini, 1967		
	*Achrysocharoidesacerianus* (Askew, 1974)		
	*Achrysocharoidesatys* (Walker, 1839)		von Heyden (1887)
	*Achrysocharoidescarpini* Bryan, 1980		
	*Achrysocharoidescilla* (Walker, 1839)		
	*Achrysocharoidescruentus* Hansson, 1983		
	*Achrysocharoideslatreillii* (Curtis, 1826)		
	*Achrysocharoidesnigricoxae* (Delucchi, 1954)		
	*Achrysocharoidesniveipes* (Thomson, 1878)		
	*Achrysocharoidespannonica* (Erdös, 1954)	CL01: *Achrysocharoidesinsignitellae* Erdös	
	*Achrysocharoidesrobiniae* Hansson & Shevtsova, 2010		
	*Achrysocharoidessplendens* (Delucchi, 1954)		von Heyden (1887)
	*Achrysocharoidessuprafolius* (Askew, 1974)		
	*Achrysocharoidesusticrus* (Erdös, 1954)	CL01: *Kratoysmausticrus* Erdös	
	*Achrysocharoideszwoelferi* (Delucchi, 1954)		
	*Allocerastichusdoderi* Masi, 1924		
	*Anaprostocetusacuminatus* (Ratzeburg, 1848)		Kopelke (1996)
	*Aprostocetusaethiops* (Zetterstedt, 1838)		
	*Aprostocetusannulatus* (Förster, 1861)		
	*Aprostocetusapama* (Walker, 1839)		
	*Aprostocetusapiculatus* Graham, 1987		
	*Aprostocetusaquaticus* (Erdös, 1954)		
	*Aprostocetusaquilus* Graham, 1987		
	*Aprostocetusarenarius* (Erdös, 1954)		
	*Aprostocetusaristaeus* (Walker, 1839)		
	*Aprostocetusartemisicola* Graham, 1987		
	*Aprostocetusboreus* (Delucchi, 1954)		
	*Aprostocetusbrachycerus* (Thomson, 1878)		
	*Aprostocetusbruzzonis* (Masi, 1930)		
	*Aprostocetuscalamarius* Graham, 1961		Schlemmermeyer (1994)
	*Aprostocetuscaudatus* Westwood, 1833		
	*Aprostocetuscecidomyiarum* (Bouché, 1834)		
	*Aprostocetusciliatus* (Nees, 1834)		
	*Aprostocetuscitrinus* (Förster, 1841)		
	*Aprostocetuscitripes* (Thomson, 1878)		
	*Aprostocetusclavicornis* (Zetterstedt, 1848)		
	*Aprostocetuscollega* (Ratzeburg, 1844)		Dorow et al. (1992)
	*Aprostocetuscrino* (Walker, 1838)		
	*Aprostocetusdiversus* (Förster, 1841)		
	*Aprostocetuselongatus* (Förster, 1841)		Hassan (1967), von Heyden (1887)
	*Aprostocetusemesa* (Walker, 1839)		
	*Aprostocetusepicharmus* (Walker, 1839)		
	*Aprostocetuseriophyes* (Taylor, 1909)		
	*Aprostocetusescherichi* (Szelènyi, 1941)		
	*Aprostocetuseupatorii* Kurdjumov, 1913		
	*Aprostocetusforsteri* (Walker, 1847)		Nitschke et al. (2017)
	*Aprostocetusfulvipes* (Förster, 1878)		
	*Aprostocetusgaus* (Walker, 1839)		
	*Aprostocetusgratus* (Giraud, 1863)		Schlemmermeyer (1994)
	*Aprostocetushagenowii* (Ratzeburg, 1852)		
	*Aprostocetusleptoneuros* (Ratzeburg, 1844)		
	*Aprostocetusleucone* (Walker, 1839)		
	*Aprostocetuslongicauda* (Thomson, 1878)		
	*Aprostocetuslongiscapus* (Thomson, 1878)		Schlemmermeyer (1994)
	*Aprostocetusluteus* (Ratzeburg, 1848)		Dorow et al. (1992)
	*Aprostocetuslycidas* (Walker, 1839)		
	*Aprostocetuslysippe* (Walker, 1839)		
	*Aprostocetusmandanis* (Walker, 1839)		
	*Aprostocetusmetra* (Walker, 1839)		
	*Aprostocetusmicroscopicus* (Rondani, 1877)		
	*Aprostocetusminimus* (Ratzeburg, 1848)		
	*Aprostocetusmycerinus* (Walker, 1839)		
	*Aprostocetusneglectus* (Domenichini, 1957)		
	*Aprostocetusnovatus* (Walker, 1839)		
	*Aprostocetusorithyia* (Walker, 1839)		Schlemmermeyer (1994)
	*Aprostocetusovivorax* (Silvestri, 1920)		
	*Aprostocetuspachyneuros* (Ratzeburg, 1844)		
	*Aprostocetuspallipes* (Dalman, 1820)		
	*Aprostocetuspausiris* (Walker, 1839)		
	*Aprostocetuspercaudatus* (Silvestri, 1920)		
	*Aprostocetusphineus* (Walker, 1839)		
	*Aprostocetusphragmiticola* Graham, 1987		Schlemmermeyer (1994)
	*Aprostocetuspseudopodiellus* (Bakkendorf, 1953)		
	*Aprostocetusptarmicae* Graham, 1987		
	*Aprostocetuspygmaeus* (Zetterstedt, 1838)		
	*Aprostocetusroesellae* (Nees, 1834)		
	*Aprostocetusrubi* Graham, 1987		
	*Aprostocetusrubicola* Graham, 1987		
	*Aprostocetusrumicis* Graham, 1987		
	*Aprostocetussalictorum* Graham, 1987		
	*Aprostocetusserratularum* Graham, 1987		
	*Aprostocetusstrobilanae* (Ratzeburg, 1844)		
	*Aprostocetussubplanus* Graham, 1987		
	*Aprostocetustanaceticola* Graham, 1987		
	*Aprostocetusterebrans* Erdös, 1954		
	*Aprostocetustorquentis* Graham, 1987		
	*Aprostocetustrjapitzini* (Kostjukov, 1976)		Hoffmann (2002)
	*Aprostocetusvassolensis* Graham, 1987		Kruess (1996)
	*Aprostocetusvenustus* (Gahan, 1914)		
	*Aprostocetusveronicae* Graham, 1987		
	*Aprostocetusxanthopus* (Nees, 1834)		
	*Aprostocetuszosimus* (Walker, 1839)		
	*Asecodescongruens* (Nees, 1834)		
	*Asecodeserxias* (Walker, 1848)		
	*Asecodeslagus* (Walker, 1838)		
	*Asecodeslucens* (Nees, 1834)		
	*Astichusarithmeticus* (Förster, 1851)		von Heyden (1887), Dorow et al. (1992)
	*Astichusmaculatus* Hedqvist, 1969		
	*Astichussolutus* Förster, 1856		von Heyden (1887)
	*Aulogymnusaceris* Förster, 1851		
	*Aulogymnusarsames* (Walker, 1838)		
	*Aulogymnuseuedoreschus* (Walker, 1839)		
	*Aulogymnusgallarum* (Linnaeus, 1761)		Hassan (1967)
	*Aulogymnusobscuripes* (Mayr, 1877)		
	*Aulogymnusskianeuros* (Ratzeburg, 1844)		
	*Baryscapusadalia* (Walker, 1839)		
	*Baryscapusagrilorum* (Ratzeburg, 1844)		
	*Baryscapusanasillus* Graham, 1991		
	*Baryscapusbonessi* Askew, 2007		
	*Baryscapusbruchophagi* (Gahan, 1913)		
	*Baryscapusconwentziae* (Ferrière, 1959)		
	*Baryscapuscrassicornis* (Erdös, 1954)		
	*Baryscapusdaira* (Walker, 1839)		
	*Baryscapusdiaphantus* (Walker, 1839)		
	*Baryscapusendemus* (Walker, 1839)		
	*Baryscapusevonymellae* (Bouché, 1834)		
	*Baryscapusgalactopus* (Ratzeburg, 1844)		
	*Baryscapusgarganus* (Domenichini, 1958)		
	*Baryscapusgradwelli* Graham, 1991		Nitschke et al. (2017)
	*Baryscapusimpeditus* (Nees, 1834)		
	*Baryscapuslotellae* (Delucchi, 1954)		
	*Baryscapusnigroviolaceus* (Nees, 1834)		
	*Baryscapusoophagus* (Otten, 1942)		
	*Baryscapuspallidae* Graham, 1991		
	*Baryscapusservadeii* (Domenichini, 1965)		
	*Baryscapusspartifoliellae* Graham, 1991		
	*Baryscapussugonjaevi* (Kostjukov, 1976)		Hoffmann (2002)
	*Baryscapusturionum* (Hartig, 1838)		
	*Ceranisusmenes* (Walker, 1839)		
	*Ceranisuspacuvius* (Walker, 1838)		
	*Chaenotetrastichusgrangeri* (Erdös, 1958)		
	*Chaenotetrastichussemiflavus* (Girault, 1917)		
	*Chrysocharisacoris* (Walker, 1839)		von Heyden (1887)
	*Chrysocharisacutigaster* Hansson, 1985		
	*Chrysocharisamasis* (Walker, 1839)		
	*Chrysocharisamyite* (Walker, 1839)		
	*Chrysocharisantoni* Hansson, 1985		
	*Chrysocharisassis* (Walker, 1839)		
	*Chrysocharisavia* Hansson, 1985		
	*Chrysocharisbudensis* (Erdös, 1954)	CL01: *Achrysocharoidesbudensis* Erdös	
	*Chrysocharischlorus* Graham, 1963		
	*Chrysocharisclarkae* Yoshimoto, 1973		
	*Chrysochariscrassiscapus* (Thomson, 1878)		
	*Chrysochariselongata* (Thomson, 1878)		
	*Chrysocharisentedonoides* (Walker, 1872)		Dorow et al. (1992)
	*Chrysocharisequiseti* Hansson, 1985		
	*Chrysochariseurynota* Graham, 1963		
	*Chrysocharisgemma* (Walker, 1839)		Neiber (2010)
	*Chrysocharisidyia* (Walker, 1839)		Greiler (1993), Hassan (1967)
	*Chrysocharisillustris* Graham, 1963		
	*Chrysocharislaomedon* (Walker, 1839)		
	*Chrysocharislaricinellae* (Ratzeburg, 1848)		
	*Chrysocharisliriomyzae* Delucchi, 1954		
	*Chrysocharismediana* Förster, 1861		Dorow et al. (1992)
	*Chrysocharisnautius* (Walker, 1846)		
	*Chrysocharisnephereus* (Walker, 1839)		Hassan (1967)
	*Chrysocharisnigricrus* (Thomson, 1878)		Hassan (1967)
	*Chrysocharisnitetis* (Walker, 1839)		Dorow et al. (1992)
	*Chrysocharisnitidifrons* Graham, 1963		
	*Chrysocharisorbicularis* (Nees, 1834)		Hassan (1967)
	*Chrysocharispallipes* (Nees, 1834)		
	*Chrysocharispentheus* (Walker, 1839)		Greiler (1993)
	*Chrysocharisphryne* (Walker, 1839)		Bachmeier (1965), von Heyden (1887)
	*Chrysocharispilosa* Delucchi, 1954		
	*Chrysocharispolyzo* (Walker, 1839)		
	*Chrysocharisprodice* (Walker, 1839)		Dorow et al. (1992), Hassan (1967)
	*Chrysocharispubens* Delucchi, 1954		
	*Chrysocharispubicornis* (Zetterstedt, 1838)		Neiber (2010)
	*Chrysocharispurpurea* Bukovskii, 1938		
	*Chrysocharissubmutica* Graham, 1963		
	*Chrysocharisviridis* (Nees, 1834)		Dorow et al. (1992), Hassan (1967)
	*Chrysonotomyiagermanica* (Erdös, 1956)		
	*Cirrospilusargei* (Crawford, 1911)		Hassan (1967)
	*Cirrospilusdiallus* Walker, 1838		Hassan (1967), von Heyden (1887)
	*Cirrospiluselegantissimus* Westwood, 1832		von Heyden (1887)
	*Cirrospiluselongatus* Bouček, 1959		
	*Cirrospiluslyncus* Walker, 1838		von Heyden (1887)
	*Cirrospiluspictus* (Nees, 1834)		von Heyden (1887)
	*Cirrospilussalatis* Walker, 1838		
	*Cirrospilussinga* Walker, 1838		
	*Cirrospilusstaryi* Bouček, 1959		
	*Cirrospilusviticola* (Rondani, 1877)		von Heyden (1887)
	*Cirrospilusvittatus* Walker, 1838		Bachmeier (1965), von Heyden (1887)
	*Closteroceruslanassa* (Walker, 1839)		
	*Closteroceruslyonetiae* (Ferrière, 1952)		
	*Closterocerusruforum* (Krausse, 1917)		Eichhorn (1992)
	*Closterocerustrifasciatus* Westwood, 1833		Neiber (2010)
	*Colpoclypeusflorus* (Walker, 1839)		
	*Crataepusmarbis* (Walker, 1839)		
	*Dahlbominusfuscipennis* (Zetterstedt, 1838)		von der Dunk (1987)
	*Dermatopeltebudensis* Erdös & Novicky, 1951		
	*Derostenusgemmeus* Westwood, 1833		Hassan (1967)
	*Derostenuspunctiscuta* Thomson, 1878		
	*Dichatomusacerinus* Förster, 1878		
	*Dicladocerusalbitarsis* Ashmead, 1900		
	*Dicladoceruseuryalus* (Haliday, 1844)		
	*Dicladoceruswestwoodii* Westwood, 1832		
	*Diglyphusbegini* (Ashmead, 1904)		
	*Diglyphuschabrias* (Walker, 1838)		Dorow et al. (1992), Hassan (1967)
	*Diglyphuscrassinervis* Erdös, 1958		
	*Diglyphusisaea* (Walker, 1838)		Greiler (1993), Hassan (1967), Dorow et al. (1992)von Heyden (1887)
	*Diglyphusminoeus* (Walker, 1838)		Greiler (1993), Hassan (1967)
	*Diglyphuspachyneurus* Graham, 1963		
	*Diglyphuspoppoea* (Walker, 1848)		Greiler (1993), Hassan (1967)
	*Diglyphuspusztensis* (Erdös & Novicky, 1951)		
	*Diglyphussubplanus* (Erdös, 1958)	CL01: *Danuviellasubplana* Erdös	
	*Dimmockiabrevicornis* (Erdös, 1954)		
	*Elachertusartaeus* (Walker, 1839)		
	*Elachertuscharondas* (Walker, 1839)		Bathon and Ruppert (1996)
	*Elachertusfenestratus* Nees, 1834		
	*Elachertusgallicus* Erdös, 1958		
	*Elachertusinunctus* Nees, 1834		Dorow et al. (1992), Hassan (1967), von Heyden (1887)
	*Elachertusisadas* (Walker, 1839)		Bachmeier (1965)
	*Elachertuslateralis* (Spinola, 1808)		von Heyden (1887)
	*Elachertuspilosiscuta* Bouček, 1971		
	*Elachertuspulcher* (Erdös, 1961)		
	*Elasmusflabellatus* (Fonscolombe, 1832)		von Heyden (1887)
	*Elasmusnudus* (Nees, 1834)		
	*Elasmusplatyedrae* Ferrière, 1935		
	*Elasmusrufiventris* Ferrière, 1947		Anton et al. (2020)
	*Elasmusschmitti* Ruschka, 1920		
	*Elasmusunicolor* (Rondani, 1877)		
	*Elasmusviridiceps* Thomson, 1878		Bachmeier (1965)
	*Elasmuswestwoodi* Giraud, 1856		
	*Entedonabdera* Walker, 1839		
	*Entedoncalcicola* Graham, 1971		
	*Entedoncioni* Thomson, 1878		
	*Entedoncionobius* Thomson, 1878		
	*Entedoncostalis* Dalman, 1820		
	*Entedoncrassiscapus* Erdös, 1944		
	*Entedondiotimus* Walker, 1839		
	*Entedonergias* Walker, 1839		
	*Entedongracilior* Graham, 1971		
	*Entedonhagenowii* (Ratzeburg, 1852)		
	*Entedonheyeri* (Ratzeburg, 1848)		Kopelke (1996)
	*Entedonincultus* Askew, 1991		
	*Entedoninsignis* Erdös, 1944		
	*Entedonlixi* Erdös, 1951		
	*Entedonlongus* Bouček, 1968		
	*Entedonmecini* Askew, 1992		
	*Entedonmethion* Walker, 1839		
	*Entedonoxys* Askew, 1991		
	*Entedonparvicalcar* Thomson, 1878	CL01: *Entedonsubovatus* Thomson	
	*Entedonphiliscus* Walker, 1851		
	*Entedonprocioni* Erdös, 1944	CL01: *Entedonmolybdaenus* Erdös	
	*Entedonpunctiscapus* Thomson, 1878		
	*Entedonrumicis* Graham, 1970		
	*Entedonsetifrons* Askew, 1991		
	*Entedontenuitarsis* Thomson, 1878		
	*Entedonthomsonianus* Erdös, 1944		
	*Entedontibialis* (Nees, 1834)		
	*Entedonzanara* Walker, 1839		
	*Entedonomphalebicolorata* (Ishii, 1933)	CL01: *Entedonastichusgaussi* Ferrière	
	*Entedonomphalecarbonaria* (Erdös, 1954)	CL01: *Entedonastichuscarbonarius* (Erdös)	
	*Euderomphalechelidonii* Erdös, 1966		
	*Euderusagrili* Bouček, 1963		Dorow et al. (1992), von Heyden (1887)
	*Euderusalbitarsis* (Zetterstedt, 1838)		Hassan (1967), Kopelke (1996)
	*Euderusviridis* Thomson, 1878		
	*Eulophusabdominalis* Nees, 1834		
	*Eulophuscyanescens* Bouček, 1958		
	*Eulophuslarvarum* (Linnaeus, 1758)		Hassan (1967), von Heyden (1887), Reder (2018)
	*Eulophuspennicornis* Nees, 1834		Hassan (1967), von Heyden (1887)
	*Eulophusramicornis* (Fabricius, 1781)		
	*Eulophussmerinthicida* Bouček, 1958		
	*Eulophusthespius* Walker, 1839		
	*Euplectrusbicolor* (Swederus, 1795)		Dorow et al. (1992), von Heyden (1887)
	*Hemiptarsenusfulvicollis* Westwood, 1833		von Heyden (1887)
	*Hemiptarsenusornatus* (Nees, 1834)		Bachmeier (1965), Dorow et al. (1992), von Heyden (1887)
	*Hemiptarsenusunguicellus* (Zetterstedt, 1838)		Dorow et al. (1992), Greiler (1993)
	*Hemiptarsenuswailesellae* Novwicki, 1929		
	*Hemiptarsenuswaterhousii* Westwood, 1833		
	*Holarcticesaclinius* (Walker, 1839)	CL01: *Grahamiaclinius* Walker	Hassan (1967)
	*Holcotetrastichusrhosaces* (Walker, 1839)		
	*Hyssopusgeniculatus* (Hartig, 1838)		von Heyden (1887)
	*Hyssopusnigritulus* (Zetterstedt, 1838)		Hassan (1967), von Heyden (1887)
	*Hyssopusolivaceus* (Thomson, 1878)		Hassan (1967), von Heyden (1887)
	*Hyssopustephridus* Yefremova, 1997		
	*Kocourekiadebilis* (Ratzeburg, 1852)		
	*Melittobiaacasta* (Walker, 1839)		Dorow et al. (1992)
	*Mestocharisbimacularis* (Dalman, 1820)		
	*Mestocharismaculata* (Förster, 1841)		
	*Microlycusbiroi* Erdös, 1951		von Heyden (1887)
	*Microlycusheterocerus* Thomson, 1878		
	*Minotetrastichusfrontalis* (Nees, 1834)		Freise and Heitland (2003)
	*Minotetrastichusplatanellus* (Mercet, 1992)		
	*Minotetrastichusprolongatus* Graham, 1987		
	*Miotropisunipuncta* (Nees, 1834)		von Heyden (1887)
	*Necremnusartynes* (Walker, 1839)		
	*Necremnuscapitatus* Bouček, 1959		von Heyden (1887)
	*Necremnuscosconius* (Walker, 1839)		
	*Necremnuscroton* (Walker, 1839)		Vogel et al. (2021)
	*Necremnusfolia* (Walker, 1839)		
	*Necremnusleucarthros* (Nees, 1834)		Hassan (1967)
	*Necremnusmetalarus* (Walker, 1839)		
	*Necremnustidius* (Walker, 1839)		Dorow et al. (1992)
	*Neochrysocharisalbiscapus* Erdös, 1954		
	*Neochrysocharisaratus* (Walker, 1838)		
	*Neochrysocharischlorogaster* (Erdös, 1966)		
	*Neochrysocharisclinias* (Walker, 1838)		
	*Neochrysochariscuprifrons* Erdös, 1954		
	*Neochrysocharisdimas* (Walker, 1839)		
	*Neochrysocharisformosus* (Westwood, 1833)		Eichhorn (1992)
	*Neochrysocharismicrostoma* (Graham, 1963)	CL01: *Omphalemicrostoma* Graham	
	*Neochrysocharisnunbergi* (Szczepanski, 1960)		
	*Omphaleacuminata* Gijswijt, 1976		
	*Omphaleaethiops* Graham, 1963		
	*Omphaleaetius* (Walker, 1839)		Lübbe (1990)
	*Omphalebetulicola* Graham, 1963		Dorow et al. (1992)
	*Omphalebrevis* Graham, 1963		
	*Omphalebreviventris* Graham, 1970		
	*Omphalechryseis* Graham, 1963		
	*Omphaleclymene* (Walker, 1839)		
	*Omphaleclypealis* (Thomson, 1878)		
	*Omphaleconnectens* Graham, 1963		
	*Omphalegrahami* Gijswijt, 1976		Dorow et al. (1992)
	*Omphaleincognita* Hansson & Shevtsova, 2012		
	*Omphaleisander* (Walker, 1839)		
	*Omphalelugens* (Nees, 1834)		Dorow et al. (1992), Hassan (1967), von Heyden (1887)
	*Omphalelugubris* Askew, 2003		
	*Omphalematrana* Erdös, 1954		
	*Omphalenitens* Graham, 1963		
	*Omphaleobscura* (Förster, 1841)	CL01: *Holcopelteobscura* Förster	
	*Omphalephruron* (Walker, 1839)		
	*Omphalerubigus* (Walker, 1839)		
	*Omphalesalicis* (Haliday, 1833)		
	*Omphalestelteri* (Bouček, 1971)	CL01: *Holcopeltestelteri* Bouček	
	*Omphalesulciscuta* (Thomson, 1878)	CL01: *Holcopeltesulciscuta* Thomson	
	*Omphaletelephe* (Walker, 1839)		
	*Omphaletheana* (Walker, 1839)	CL01: *Omphaleradialis* Thomson	
	*Omphalevaripes* (Thomson, 1878)		
	*Omphaleversicolor* (Nees, 1834)		
	*Oomyzusgalerucivorus* (Hedqvist, 1959)		Meiners (1999)
	*Oomyzusgallerucae* (Fonscolombe, 1832)		
	*Oomyzusincertus* (Ratzeburg, 1844)		
	*Oomyzuspegomyae* Graham, 1991		
	*Oomyzusscaposus* (Thomson, 1878)		Triltsch (1997)
	*Oomyzussempronius* (Erdös, 1954)		
	*Parasecodellaobscura* (Thomson, 1878)		
	*Pediobiusalaspharus* (Walker, 1839)		Dorow et al. (1992)
	*Pediobiusalcaeus* (Walker, 1839)		Dorow et al. (1992)
	*Pediobiusbrachycerus* (Thomson, 1878)		
	*Pediobiuscalamagrostidis* Dawah, 1988		Dorow et al. (1992)
	*Pediobiuscassidae* Erdös, 1958		
	*Pediobiuschilaspidis* Bouček, 1965		
	*Pediobiusclaridgei* Dawah, 1988		Neugebauer (1997)
	*Pediobiusclita* (Walker, 1839)		von Heyden (1887)
	*Pediobiuscrassicornis* (Thomson, 1878)		Hassan (1967)
	*Pediobiusdactylicola* Dawah, 1988		Greiler (1993)
	*Pediobiusepeus* (Walker, 1839)		Dorow et al. (1992)
	*Pediobiusepigonus* (Walker, 1839)		Hassan (1967)
	*Pediobiuseubius* (Walker, 1839)		Greiler (1993), Hassan (1967), von Heyden (1887)
	*Pediobiusfacialis* (Giraud, 1863)		
	*Pediobiusfestucae* Dawah, 1988		Dorow et al. (1992)
	*Pediobiusflaviscapus* (Thomson, 1878)		
	*Pediobiusfoliorum* (Geoffroy, 1785)		
	*Pediobiuslysis* (Walker, 1839)		Hassan (1967)
	*Pediobiusmetallicus* (Nees, 1834)		Greiler (1993), Hassan (1967), von Heyden (1887), Neiber (2010)
	*Pediobiusnigritarsis* (Thomson, 1878)		
	*Pediobiusphalaridis* Dawah, 1988		
	*Pediobiusphragmitis* Bouček, 1965		
	*Pediobiusphyllotretae* (Riley, 1884)		
	*Pediobiusplaniventris* (Thomson, 1878)		
	*Pediobiuspolanensis* Bouček, 1965		
	*Pediobiuspyrgo* (Walker, 1839)		
	*Pediobiussaulius* (Walker, 1839)		Kopelke (1996), von Heyden (1887)
	*Pediobiustenuicornis* Graham, 1993		S. Schmidt, pers. obs.
	*Pediobiustermerus* (Walker, 1839)		
	*Pediobiustetratomus* (Thomson, 1878)		
	*Platyplectruschlorocephalus* (Nees, 1834)		
	*Platyplectruslaeviscuta* (Thomson, 1878)		
	*Platyplectruspannonica* (Erdös, 1966)		
	*Pnigalioagraules* (Walker, 1839)		Hassan (1967), von Heyden (1887), Freise and Heitland (2003)
	*Pnigaliocristatus* (Ratzeburg, 1848)		
	*Pnigalioepilobii* Bouček, 1966		
	*Pnigaliolongulus* (Zetterstedt, 1838)		von Heyden (1887)
	*Pnigaliomonilicornis* (Zetterstedt, 1838)		
	*Pnigalionemati* (Westwood, 1838)		Kopelke (1996)
	*Pnigaliopectinicornis* (Linnaeus, 1758)		Bachmeier (1965), Greiler (1993), Bachmeier (1965)
	*Pnigaliophragmitis* (Erdös, 1954)		
	*Pnigaliosoemius* (Walker, 1839)		von Heyden (1887)
	*Pnigaliotricuspis* (Erdös, 1954)		
	*Pnigaliotridentatus* (Thomson, 1878)		
	*Pronotaliacarlinarum* (Szelènyi & Erdös, 1951)		Nitschke et al. (2017)
	*Pronotaliainflata* Graham, 1991		
	*Pronotaliatrypetae* Gradwell, 1957		Nitschke et al. (2017)
	*Quadrastichusmisellus* (Delucchi, 1954)		
	*Quadrastichuspedicellaris* (Thomson, 1878)		
	*Quadrastichussajoi* (Szelènyi, 1941)		
	*Rhicnopeltecrassicornis* (Nees, 1834)		
	*Sigmophorabrevicornis* (Panzer, 1804)		
	*Sphenolepispygmaea* Nees, 1834		
	*Stenomesiusrufescens* (Retzius, 1783)		von Heyden (1887)
	*Stepanoviaaurantiaca* (Ratzeburg, 1852)	CL01: *Aprostocetusaurantiacus* Ratzeburg	
	*Stepanoviaeurytomae* (Nees, 1834)	CL01: *Aprostocetuseurytomae* Nees	
	*Sympiesisacalle* (Walker, 1848)		von Heyden (1887)
	*Sympiesisdolichogaster* Ashmead, 1888		
	*Sympiesiseuspilapterygis* (Erdös, 1958)		
	*Sympiesisflavopicta* Bouček, 1959		
	*Sympiesisgordius* (Walker, 1839)		Dorow et al. (1992), von Heyden (1887)
	*Sympiesisgrahami* Erdös, 1966		
	*Sympiesisgregori* Bouček, 1959		
	*Sympiesiskelebiana* Erdös, 1966		
	*Sympiesislucida* Storozheva, 1981		
	*Sympiesisnotata* (Zetterstedt, 1838)		
	*Sympiesissericeicornis* (Nees, 1834)		von Heyden (1887)
	*Sympiesissolitaria* Szelényi, 1977		
	*Sympiesisviridula* (Thomson, 1878)		
	*Sympiesisxanthostoma* (Nees, 1834)		
	*Tamarixiaactis* (Walker, 1839)		
	*Tamarixiamonesus* (Walker, 1839)		
	*Tamarixiapronomus* (Walker1839)		
	*Tamarixiapubescens* (Nees, 1834)		
	*Tamarixiaupis* (Walker, 1839)		Dorow et al. (1992)
	*Tetrastichusacutiusculus* (Graham, 1961)		
	*Tetrastichusagrilocidus* Graham, 1991		Hansson and Schmidt (2020)
	*Tetrastichusasilis* Hansson & Schmidt, 2020		Hansson and Schmidt (2020)
	*Tetrastichusatratulus* (Nees, 1834)		Hansson and Schmidt (2020)
	*Tetrastichusclito* (Walker, 1840)		Hansson and Schmidt (2020)
	*Tetrastichuscoelarchus* Graham, 1991		
	*Tetrastichuscoeruleus* (Nees, 1834)		Hansson and Schmidt (2020)
	*Tetrastichusdelvarei* Hansson & Schmidt, 2019		Hansson and Schmidt (2020)
	*Tetrastichusdoczkali* Hansson & Schmidt, 2020		Hansson and Schmidt (2020)
	*Tetrastichuselodius* Hansson & Schmidt, 2020		Hansson and Schmidt (2020)
	*Tetrastichushalidayi* (Graham, 1961)		Hansson and Schmidt (2020)
	*Tetrastichusheeringi* Delucchi, 1954		Hansson and Schmidt (2020)
	*Tetrastichushylotomarum* (Bouché, 1834)		Hansson and Schmidt (2020)
	*Tetrastichusilithyia* (Walker, 1839)		Hansson and Schmidt (2020)
	*Tetrastichusillydris* Hansson & Schmidt, 2020		Hansson and Schmidt (2020)
	*Tetrastichusjulis* (Walker, 1839)		Hansson and Schmidt (2020)
	*Tetrastichuslegionarius* Giraud, 1863		
	*Tetrastichuslyridice* (Walker, 1839)		
	*Tetrastichusmiser* (Nees, 1834)		Hansson and Schmidt (2020)
	*Tetrastichusmurcia* (Walker, 1839)		Hansson and Schmidt (2020)
	*Tetrastichuspolyporinus* Askew, 2007		
	*Tetrastichussetifer* Thomson, 1878		
	*Tetrastichussinope* (Walker, 1839)		
	*Tetrastichustacitus* Hansson & Schmidt, 2020		Hansson and Schmidt (2020)
	*Tetrastichustelon* (Graham, 1961)		Hansson and Schmidt (2020)
	*Tetrastichusulmi* Erdös, 1954		Hansson and Schmidt (2020)
	*Trjapitzinichusevanescens* (Ratzeburg, 1848)	CL01: *Aceratonuromyiaevanescens* Ratzeburg	
	*Xanthellaszabopatayi* Moczar, 1950	CL01: *Xanthellumtranssylvanicum* Erdös	von Heyden (1887)
**Eupelmidae**			
	*Anastatusbifasciatus* (Geoffroy, 1785)		
	*Anastatuscatalonicus* Bolivar, y, Pieltain, 1935		Anton et al. (2020)
	*Anastatusgiraudi* (Ruschka, 1921)		
	*Anastatusjaponicus* Ashmead, 1904		
	*Anastatusoscari* (Ruthe, 1859)		
	*Brasemastenus* (Bouček, 1968)	CL01: *Eupelmusstenus* Bouček	
	*Calosotaacron* Ruschka, 1921		von Heyden (1887)
	*Calosotaaestivalis* Curtis, 1836		
	*Calosotametallica* (Gahan, 1922)	CL01: *Calosotaviridis* Masi	
	*Calosotaobscura* Ruschka, 1921		
	*Calosotavernalis* Curtis, 1836		von Heyden (1887), Anton et al. (2020)
	*Calymmochilussubnubilus* (Walker, 1872)		
	*Eupelmusannulatus* Nees, 1834		Dorow et al. (1992), von Heyden (1887)
	*Eupelmusatropurpureus* Dalman, 1820		
	*Eupelmusazureus* Ratzeburg, 1844		
	*Eupelmusconfusus* Al Khatib, 2015		Anton et al. (2020)
	*Eupelmusfulvipes* Förster, 1860		
	*Eupelmusfuscipennis* Förster, 1860		
	*Eupelmushartigi* Förster, 1841		
	*Eupelmuskiefferi* De Stefani, 1898		
	*Eupelmuslinearis* Förster, 1860		
	*Eupelmuslongicalvus* Al Khatib & Fusu, 2015		
	*Eupelmusmessene* Walker, 1839		
	*Eupelmusmicrozonus* Förster, 1860		
	*Eupelmusopacus* Delvare, 2015		
	*Eupelmusphragmitis* Erdös, 1955		
	*Eupelmuspini* Taylor, 1927		
	*Eupelmussimizonus* Al Khatib, 2015		
	*Eupelmussplendens* Giraud, 1872		
	*Eupelmusstramineipes* Nikol'skaya, 1952		
	*Eupelmusurozonus* Dalman, 1820	CL01: *Eupelmusbedeguaris* Ratzeburg; *E.hostilis* Förster	Dorow et al. (1992), Greiler (1993), Hassan (1967), Kopelke (1996)
	*Eupelmusvesicularis* (Retzius, 1783)		Dorow et al. (1992), Kopelke (1996), Neugebauer (1997), von Heyden (1887), Nitschke et al. (2017)
	*Eusandalumcoronatum* (Thomson, 1876)		
	*Eusandalumelongatum* (Ruschka, 1921)		
	*Eusandalumflavipenne* Ruschka, 1921		
	*Eusandaluminerme* (Ratzeburg, 1848)		
	*Eusandalummerceti* (Bolivar y Pieltain, 1926)		Anton et al. (2020)
	*Eusandalumwalkeri* (Curtis, 1836)		
	*Merostenusexcavatus* (Dalman, 1820)		
	*Merostenushungaricus* (Erdös, 1959)		
	*Merostenusrostratus* (Ruschka, 1921)	CL01: *Eupelmusrostratus* Ruschka	
	*Metapelmanobile* (Förster, 1860)	CL01: *Metapelmanobilis* Förster	Anton et al. (2020)
**Eurytomidae**			
	*Aximopsisnodularis* (Boheman, 1836)	CL01: *Eurytomanodularis* Boheman	
	*Bruchophagusastragali* Fedoseeva, 1954		
	*Bruchophagusater* (Walker, 1832)	CL01: *Eurytomaatra* Walker	
	*Bruchophagusgibbus* (Boheman, 1836)		Kruess (1996), Anton et al. (2020)
	*Bruchophagusphlei* (Erdös, 1969)		Neugebauer (1997)
	*Bruchophagusplatypterus* (Walker, 1834)		Anton et al. (2020)
	*Bruchophagusroddi* Gussakovskiy, 1933		Anton et al. (2020)
	*Eurytomaaciculata* Ratzeburg, 1848		
	*Eurytomaadleriae* Zerova, 1995		
	*Eurytomaafra* Boheman, 1836	CL01: *Eurytomaspessivtsevi* Bouček & Novicky	
	*Eurytomaaloineae* (Burks, 1958)		
	*Eurytomaappendigaster* (Swederus, 1795)		
	*Eurytomaaquatica* Erdös, 1955		
	*Eurytomaarctica* Thomson, 1876	CL01: *Eurytomablastophagi* Heqvist	
	*Eurytomaaspila* (Walker, 1836)		Anton et al. (2020)
	*Eurytomabaldingerae* Erdös, 1961		
	*Eurytomabrunniventris* Ratzeburg, 1852		
	*Eurytomacastor* Claridge, 1959		Hassan (1967)
	*Eurytomacaulicola* Zerova, 1971		
	*Eurytomacentaureae* Claridge, 1960		
	*Eurytomacollaris* Walker, 1832		
	*Eurytomacompressa* (Fabricius, 1794)		Nitschke et al. (2017)
	*Eurytomacoxalis* Erdös, 1969		
	*Eurytomacrassinervis* Thomson, 1876		Schlemmermeyer (1994)
	*Eurytomacurculionum* Mayr, 1878		Thies et al. (1997)
	*Eurytomacurta* Walker, 1832		
	*Eurytomacynipsea* Boheman, 1836		Anton et al. (2020)
	*Eurytomadanilovi* Zerova, 1985		
	*Eurytomadanuvica* Erdös, 1955		Dorow et al. (1992)
	*Eurytomadentata* Mayr, 1878		
	*Eurytomaerdoesi* Szelényi, 1974		
	*Eurytomaflavimana* Boheman, 1836		
	*Eurytomagoidanichi* Bouček, 1970		
	*Eurytomahypochoeridis* Claridge, 1960		
	*Eurytomajaceae* Mayr, 1878		
	*Eurytomakangasi* Hedqvist, 1966		
	*Eurytomamaura* (Boheman, 1836)	CL01: *Bruchophagusmaura* Boheman	
	*Eurytomamayri* Ashmead, 1877		
	*Eurytomamorio* Boheman, 1836	CL01: *Eurytomaeccoptogastri* Ratzeburg; *E.flavoscapularis* Ratzeburg; *E.flavovaria* Ratzeburg	
	*Eurytomanobbei* Mayr, 1878		
	*Eurytomaobscura* Boheman, 1836		
	*Eurytomaonobrychidis* Nikol'skaya, 1933		
	*Eurytomaoophaga* Silvestri, 1920		
	*Eurytomapediaspisi* Pujade i Villar, 1994		
	*Eurytomaphalaridis* Graham, 1974		
	*Eurytomapistaciae* Rondani, 1877		
	*Eurytomapollux* Claridge, 1959		Hassan (1967)
	*Eurytomarobusta* Mayr, 1878		
	*Eurytomarosae* Nees, 1834		
	*Eurytomaroseni* Claridge, 1959		
	*Eurytomarufa* Zerova, 1970		
	*Eurytomarufipes* Walker, 1832		
	*Eurytomaserratulae* (Fabricius, 1798)		
	*Eurytomasetigera* Mayr, 1878		
	*Eurytomastrigifrons* Thomson, 1876		
	*Eurytomastriolata* Ratzeburg, 1848		
	*Eurytomatapio* Claridge, 1959		
	*Eurytomatruncatella* Zerova, 1978		
	*Eurytomaverticillata* (Fabricius, 1798)		Anton et al. (2020)
	*Eurytomawachtli* Mayr, 1878		
	*Mangomasalicis* (Walker, 1834)		
	*Sycophilabiguttata* (Swederus, 1795)		
	*Sycophilaconcinna* (Boheman, 1836)		
	*Sycophilafasciata* (Thomson, 1876)	CL01: *Sycophilastagnalis* Erdös	
	*Sycophilaflavicollis* (Walker, 1834)		Thies et al. (1997)
	*Sycophilamellea* (Curtis, 1831)		Dorow et al. (1992)
	*Sycophilasubmutica* (Thomson, 1876)		Bathon and Ruppert (1996)
	*Systolealbipennis* Walker, 1832		
	*Systoleatratula* (Dalla, Torre, 1898)		
	*Tetramesaaciculata* (Schlechtendal, 1891)		
	*Tetramesaaffinis* (Hedicke, 1921)		
	*Tetramesaagrostidis* (Howard, 1896)		
	*Tetramesaairae* (Schlechtendal, 1891)		Hassan (1967)
	*Tetramesaalbomaculatum* (Ashmead, 1894)		
	*Tetramesabrachypodii* (Schlechtendal, 1891)		
	*Tetramesabrevicollis* (Walker, 1836)		
	*Tetramesabrevicornis* (Walker, 1832)		
	*Tetramesabrischkei* (Schlechtendal, 1891)		
	*Tetramesacalamagrostidis* (Schlechtendal, 1891)		
	*Tetramesacylindrica* (Schlechtendal, 1891)		
	*Tetramesaeximia* (Giraud, 1863)		Dorow et al. (1992)
	*Tetramesafoersteri* (Hedicke, 1921)		
	*Tetramesafulvicollis* (Walker, 1832)		
	*Tetramesafumipennis* (Walker, 1832)		
	*Tetramesagiraudi* (Schlechtendal, 1891)		Hassan (1967)
	*Tetramesahordei* (Harris, 1830)		
	*Tetramesahyalipennis* (Walker, 1832)		
	*Tetramesalaevigata* (Hedicke, 1921)		
	*Tetramesalinearis* (Walker, 1832)		
	*Tetramesalongicornis* (Walker, 1832)		
	*Tetramesalongula* (Dalman, 1820)		
	*Tetramesamaritima* (Hedicke, 1921)		
	*Tetramesanovalis* Zerova, 1978		
	*Tetramesapetiolata* (Walker, 1832)		Hassan (1967)
	*Tetramesaphleicola* (Hedicke, 1921)		
	*Tetramesaphragmitis* (Erdös, 1952)		
	*Tetramesapoae* (Schlechtendal, 1891)		
	*Tetramesapuccinellae* Zerova, 1976		
	*Tetramesascheppigi* (Schlechtendal, 1891)		
	*Tetramesaschlechtendali* (Hedicke, 1921)		
	*Tetramesaschmidti* (Hedicke, 1921)		
**Leucospidae**			
	*Leucospisdorsigera* Fabricius, 1775		von Heyden (1887), Reder (2015), Reder (2005)
	*Leucospisgigas* Fabricius, 1793		
	*Leucospisintermedia* Illiger, 1807		
**Megastigmidae**			
	*Bootanomyiadorsalis* (Fabricius, 1798)	CL01: *Megastigmusdorsalis* Fabricius	
	*Bootanomyiastigmatizans* (Fabricius, 1798)	CL01: *Megastigmusstigmatizans* Fabricius	
	*Megastigmusaculeatus* (Swederus, 1795)		Anton et al. (2020)
	*Megastigmusatedius* Walker, 1851		
	*Megastigmusbipunctatus* (Swederus, 1795)		
	*Megastigmusbrevicaudis* Ratzeburg, 1852		
	*Megastigmuspictus* (Förster, 1841)	CL01: *Megastigmusseitneri* Hoffmeyer	Anton et al. (2020)
	*Megastigmuspinus* Parfitt, 1857		
	*Megastigmusrosae* Bouček, 1971		
	*Megastigmusspermotrophus* Wachtl, 1893		
	*Megastigmusstrobilobius* Ratzeburg, 1848		
	*Megastigmussuspectus* Borries, 1895		
**Mymaridae**			
	*Alaptusfusculus* Walker, 1846	CL01: *Alaptusextremus* Soyka; *A.novickyi* Soyka	Müller and Triapitsyn (2020)
	*Alaptusminimus* Westwood, 1839	CL01: *Alaptusauranti* Mercet; *A.malachinensis* Soyka	Triapitsyn et al. (2020), Anton et al. (2020), Müller and Triapitsyn (2020)
	*Alaptuspallidicornis* Förster, 1856		Anton et al. (2020), Müller (2020)
	*Alaptusstammeri* Soyka, 1939		
	*Anagrusatomus* (Linnaeus, 1767)		
	*Anagrusbakkendorfi* Sokya, 1946	CL01: *Anagrusavalae* Soyka	Böll et al. (1999)
	*Anagrusensifer* Debauche, 1948		
	*Anagrusincarnatosimilis* Soyka, 1956		
	*Anagrusincarnatus* Haliday, 1833	CL01: *Anagrusbreviphragma* Soyka	
	*Anagrusnigriceps* (Smits, van, Burgst, 1914)	CL01: *Anagrussimilis* Soyka	
	*Anagrusoptabilis* (Perkins, 1905)		Müller and Triapitsyn (2020)
	*Anagrussubfuscus* Förster, 1847	CL01: *Anagrussupremosimilis* Soyka	
	*Anaphesbrevis* Walker, 1846	CL01: *Anaphesmalchinensis* Soyka	
	*Anaphescollinus* Walker, 1846	CL01: *Anaphesdiscolorisimilis* Soyka	
	*Anaphescrassicornis* (Walker, 1846)	CL01: *Anaphesvariatus* Soyka	
	*Anaphescrassipennis* (Soyka, 1946)	CL01: *Anaphesaries* Debauche; *A.gracillimus* Soyka; *A.serenus* Soyka; *A.sulphuripes* Soyka; *A.wertaneki* Soyka	
	*Anaphesdiana* (Girault, 1911)	CL01: *Anaphescompressus* Soyka	Müller and Triapitsyn (2020)
	*Anaphesflavipes* (Förster, 1841)	CL01: *Anaphesgauthieri* Debauche; *A.neospecialis* Soyka; *A.pilicornis* Soyka	
	*Anaphesfuscipennis* Haliday, 1833		
	*Anaphesgauthieri* Debauche, 1948	CL01: *Anaphesflavus* Soyka	
	*Anapheslongicornis* Walker, 1846	CL01: *Anaphesleptoceras* Debauche; *A.pannonicus* Soyka	
	*Anaphesluna* (Girault, 1914)	CL01: *Anaphesbrachygaster* Debauche; *A.dorcas* Debauche	
	*Anaphesmedius* Soyka, 1946	CL01: *Anaphesintermedius* Soyka	Müller and Triapitsyn (2020)
	*Anaphesovipositor* Soyka, 1946	CL01: *Anaphesbrevitarsis* Soyka; *A.angustipennis* Soyka	
	*Anaphesparallelipennis* (Soyka, 1949)	CL01: *Anaphesrectipennis* Soyka	
	*Anaphesquadraticornis* (Soyka, 1949)		
	*Anaphesregulus* Walker, 1846		
	*Anaphessilesicus* (Soyka, 1946)	CL01: *Anaphesexiguus* Soyka	
	*Arescondimidiatus* (Curtis, 1832)	CL01: *Arescondimidiata*	Müller and Triapitsyn (2020),
	*Camptopteracardui* (Förster, 1856)	CL01: *Camptopteraaula* Debauche; *C.stammeri* Soyka; *C.tenuis* Soyka	Müller and Triapitsyn (2020)
	*Camptopteramagna* Soyka, 1946		
	*Camptopterapapaveris* Förster, 1856	CL01: *Camptopteraannulata* Soyka; *C.parva* Soyka	Müller and Triapitsyn (2020)
	*Camptopterapunctum* (Shaw, 1798)	CL01: *Camptopteraellifranzae* Strassen	
	*Cleruchusjanetscheki* Novicky, 1965		
	*Cleruchuspluteus* Enock, 1909		
	*Cosmocomoideaatra* (Förster, 1841)	CL01: *Gonatocerusater*	Anton et al. (2020), Müller and Triapitsyn (2020)
	*Cosmocomoideaoxypygus* (Foerster, 1856)		
	*Erythmelusagilis* (Enock, 1909)		Müller and Triapitsyn (2020)
	*Erythmelusdichromocnemus* Nowicki, 1953		Müller and Triapitsyn (2020)
	*Erythmelusflavovarius* (Walker, 1846)	CL01: *Erythmelusgoochi* Enock	
	*Erythmeluspanis* (Enock, 1909)		
	*Erythmelusrex* (Girault, 1911)		
	*Eustochusatripennis* (Curtis, 1832)		Müller and Triapitsyn (2020)
	*Eustochuscinctus* (Walker, 1846)		Triapitsyn et al. (2020)
	*Eustochusconfusus* Huber & Baquero, 2007		Müller and Triapitsyn (2020)
	*Eustochuskoponeni* Triapitsyn, 2020		
	*Gonatocerusfuscicornis* (Walker, 1846)	CL01: *Gonatocerussulphuripes* (Förster)	Müller and Triapitsyn (2020)
	*Gonatoceruslongicornis* Nees, 1834		Anton et al. (2020), Müller and Triapitsyn (2020)
	*Gonatoceruspictus* (Haliday, 1833)		Müller and Triapitsyn (2020)
	*Lituscynipseus* Haliday, 1833		Anton et al. (2020), Müller (2020)
	*Lymaenonacuminatus* Walker, 1846		
	*Lymaenonaureus* (Girault, 1911)		Müller and Triapitsyn (2020)
	*Lymaenonlitoralis* (Haliday, 1833)	CL01: *Gonatoceruslitoralis* Soyka	Anton et al. (2020), Müller and Triapitsyn (2020)
	*Lymaenonlongior* (Soyka, 1946)	CL01: *Gonatoceruslongior* Soyka	
	*Mymarpulchellum* Curtis, 1832		Müller and Triapitsyn (2020)
	*Mymarregale* Enock, 1911		Müller and Triapitsyn (2020)
	*Mymartaprobanicum* Ward, 1875		Müller and Triapitsyn (2020)
	*Ooctonushemipterus* Haliday, 1833	CL01: *Ooctonuswagneri* Soyka	Müller and Triapitsyn (2020)
	*Ooctonusinsignis* Haliday, 1833	CL01: *Ooctonusmajor* Girault	
	*Ooctonusnotatus* Walker, 1846		Anton et al. (2020), Müller and Triapitsyn (2020)
	*Ooctonusnovickyi* Soyka, 1950		Müller and Triapitsyn (2020)
	*Ooctonussublaevis* Förster, 1847		Anton et al. (2020)
	*Ooctonusvulgatus* Haliday, 1833		Müller and Triapitsyn (2020)
	*Polynemaatratum* Haliday, 1833		Müller and Triapitsyn (2020)
	*Polynemacapillatum* Soyka, 1956		
	*Polynemacrassicorne* Förster, 1847		
	*Polynemaeuchariforme* Haliday, 1833		Müller and Triapitsyn (2020)
	*Polynemaflavipes* Walker, 1846		
	*Polynemafoersteri* Soyka, 1946		
	*Polynemafumipenne* Walker, 1846		Müller and Triapitsyn (2020)
	*Polynemafuscipes* Haliday, 1833		
	*Polynemagracile* (Nees, 1834)		
	*Polynemagracilior* Soyka, 1946		
	*Polynemalaetum* Förster, 1847		
	*Polynemalatipenne* Förster, 1847		
	*Polynemamalkwitzi* Soyka, 1956		
	*Polynemamarginatum* (Soyka, 1940)		
	*Polynemaneustadti* Soyka, 1956		
	*Polynemaovatum* Soyka, 1956		
	*Polynemaovulorum* (Linnaeus, 1758)		
	*Polynemapusillum* Haliday, 1833		
	*Polynemasachtlebeni* Soyka, 1956		
	*Polynemaschmitzi* Soyka, 1956		
	*Polynemaspectabile* (Soyka, 1956)		
	*Polynemastammeri* Soyka, 1946		
	*Polynemavitripenne* (Förster, 1847)		
	*Stephanodessimilis* (Förster, 1847)		
	*Stethyniumtriclavatum* Enock, 1909		Böll et al. (1999)
**Ormyridae**			
	*Ormyruscingulatus* (Förster, 1860)		
	*Ormyrusdiffinis* (Fonscolombe, 1832)		
	*Ormyrusgratiosus* (Förster, 1860)		Bathon and Ruppert (1996), Anton et al. (2020)
	*Ormyrusnitidulus* (Fabricius, 1804)		
	*Ormyrusorientalis* Walker, 1871		Nitschke et al. (2017)
	*Ormyruspapaveris* (Perris, 1840)		
	*Ormyruspomaceus* (Geoffroy, 1785)		
	*Ormyrusrufimanus* Mayr, 1904		Schmidt, pers. comm.
	*Ormyruswachtli* Mayr, 1904		
**Perilampidae**			
	*Chrysolampuspunctatus* (Förster, 1859)		
	*Chrysolampusrufitarsis* (Förster, 1859)		
	*Chrysolampussplendidulus* (Spinola, 1808)		
	*Chrysolampusthenae* (Walker, 1848)		
	*Chrysomallaroseri* Förster, 1859		
	*Perilampusaeneus* (Rossius, 1790)		Anton et al. (2020)
	*Perilampusauratus* (Panzer, 1798)		
	*Perilampusaureoviridis* Walker, 1833		
	*Perilampuschrysonotus* Förster, 1859		Agricola (1984)
	*Perilampusintermedius* Bouček, 1956		
	*Perilampuslaevifrons* Dalman, 1822		Agricola (1984)
	*Perilampusmasculinus* Bouček, 1956		
	*Perilampusmicans* Dalman, 1820		von Heyden (1887)
	*Perilampusminutalis* Steffan, 1952		
	*Perilampusneglectus* Bouček, 1956		
	*Perilampusnitens* Walker, 1834		von Heyden (1887)
	*Perilampusruficornis* (Fabricius, 1793)		
	*Perilampusruschkai* Hellén, 1924		
	*Perilampustristis* Mayr, 1905		Agricola (1984)
**Pteromalidae**			
	*Ablaxiarobusta* Hedqvist, 1978		
	*Acrocormussemifasciatus* Thomson, 1878		Haas et al. (2021)
	*Aggelmaviolacea* (Zetterstedt, 1838)		
	*Anisopteromaluscalandrae* (Howard, 1881)		
	*Anogmushohenheimensis* Ratzeburg, 1844		
	*Anogmushungaricus* (Erdös, 1948)		
	*Anogmuspiceae* (Ruschka, 1921)		
	*Anogmusstrobilorum* (Thomson, 1878)		
	*Anogmusvala* (Walker, 1839)		
	*Apeliomapteromalinum* (Thomson, 1878)		Haas et al. (2021)
	*Apsilocerabramleyi* Graham, 1966		
	*Arthrolytusdiscoideus* (Nees, 1834)		
	*Arthrolytusmaculipennis* (Walker, 1835)		
	*Arthrolytusocellus* (Walker, 1834)		
	*Arthrolytusslovacus* Graham, 1969		Haas et al. (2021)
	*Asaphessuspensus* (Nees, 1834)		Blüthgen (1929)
	*Asaphesvulgaris* Walker, 1834		Blüthgen (1929), Anton et al. (2020)
	*Atrichomalustrianellatus* Graham, 1956		Haas et al. (2021)
	*Caenacisinflexa* (Ratzeburg, 1848)		
	*Caenacislauta* (Walker, 1835)		
	*Caenocrepisarenicola* (Thomson, 1878)		
	*Callitulabicolor* Spinola, 1811		
	*Callitulaelongata* (Thomson, 1878)		
	*Callitulaferrierei* Bouček, 1964		
	*Callitulapyrrhogaster* (Walker, 1833)		
	*Capelliacecidomyiae* (Ratzeburg, 1844)		
	*Capelliaorneus* (Walker, 1839)		
	*Catolaccusater* (Ratzeburg, 1852)		
	*Ceapulicaris* Walker, 1837		Anton et al. (2020)
	*Cecidolampabarbotini* Askew, 1975		
	*Cecidostibadocimus* (Walker, 1839)		
	*Cecidostibafungosa* (Geoffroy, 1785)		
	*Cecidostibageganius* (Walker, 1848)		
	*Cecidostibajucundus* (Förster, 1841)		Wall (1973)
	*Cecidostibasemifascia* (Walker, 1835)		
	*Cerocephalacornigera* Westwood, 1832		
	*Cerocephalarufa* (Walker, 1833)		
	*Cheiropachusquadrum* (Fabricius, 1787)		Anton et al. (2020), Greiler (1993), von Heyden (1887)
	*Chlorocytusalticornis* Graham, 1984		
	*Chlorocytusbreviscapus* Graham, 1965		Dorow et al. (1992)
	*Chlorocytusdiversus* (Walker, 1836)		
	*Chlorocytusformosus* (Walker, 1835)		
	*Chlorocytusharmolitae* Bouček, 1957		Blüthgen (1929)
	*Chlorocytusinchoatus* Graham, 1965		
	*Chlorocytuslongicauda* (Thomson, 1878)		
	*Chlorocytusphalaridis* Graham, 1965		
	*Chlorocytuspolichna* (Walker, 1848)		
	*Chlorocytusspicatus* (Walker, 1835)		Lübbe (1990)
	*Chlorocytusterminalis* (Walker, 1839)		
	*Chlorocytusultonicus* Graham, 1965		
	*Cleonymusbrevis* Bouček, 1972		Haas et al. (2021)
	*Cleonymuslaticornis* Walker, 1837		
	*Cleonymusobscurus* Walker, 1837		Haas et al. (2021)
	*Cleonymusvrevis* Bouček, 1972		
	*Coelopisthiaareolata* Askew, 1980	CL01: *Kranophorusareolata* Askew	
	*Coelopisthiacaledonica* Askew, 1980		
	*Coelopisthiaextenta* (Walker, 1835)	CL01: *Kranophorusextentus* Walker	Dorow et al. (1992), Anton et al. (2020)
	*Coelopisthiapachycera* Masi, 1934		Haas et al. (2021)
	*Collentissuecicus* Graham, 1969	CL01: *Callimerismussuecicus* Graham	
	*Colotrechnussubcoeruleus* Thomson, 1878		
	*Conomoriumamplum* (Walker, 1835)		
	*Conomoriumpatulum* (Walker, 1835)		
	*Corunaclavata* Walker, 1833		Blüthgen (1929)
	*Cratomusmegacephalus* (Fabricius, 1793)		
	*Cryptoprymnaatra* (Walker, 1833)		
	*Cryptoprymnapaludicola* Askew, 1991		Haas et al. (2021)
	*Cyclogastrellaclypealis* Bouček, 1965		Haas et al. (2021)
	*Cyclogastrellasimplex* (Walker, 1834)		
	*Cyrtogasterclavicornis* Walker, 1833		
	*Cyrtogastervulgaris* Walker, 1833		Greiler (1993), Neiber (2010)
	*Dibrachoidescionobius* Graham, 1969		Anton et al. (2020)
	*Dibrachoidesdynastes* (Förster, 1841)		
	*Dibrachysaffinis* Masi, 1907		
	*Dibrachysfuscicornis* (Walker, 1836)		
	*Dibrachyshians* Bouček, 1965		Haas et al. (2021)
	*Dibrachyslignicola* Graham, 1969	CL01: *Dibrachysgoettingenus* Doganlar	
	*Dibrachysmicrogastri* (Bouché, 1834)	CL01: *Dibrachysboarmiae* Walker; *D.cavus* Walker; *D.vesparum* Ratzeburg	Bachmeier (1965)
	*Dibrachysverovesparum* Peters & Baur, 2011		
	*Diglochispaludicola* Abraham, 1986		
	*Diglochissylvicola* (Walker, 1835)		
	*Dimachuscingulum* (Nees, 1834)		
	*Dinarmusacutus* (Thomson, 1878)		
	*Dinotiscusaponius* (Walker, 1848)		
	*Dinotiscusavrupanensis* Doganlar, 2007		
	*Dinotiscuscalcaratus* (Thomson, 1878)		
	*Dinotiscuscolon* (Linnaeus, 1758)		
	*Dinotiscuseupterus* (Walker, 1836)		
	*Dinotiscusisvicrensis* Doganlar, 2007		
	*Dinotoidestenebricus* (Walker, 1834)		Haas et al. (2021)
	*Diparapetiolata* Walker, 1833		
	*Dirhicnusramealis* (Nees, 1834)		
	*Ecrizoteslongicornis* (Walker, 1848)		Haas et al. (2021)
	*Ecrizotesmonticola* Förster, 1861		Haas et al. (2021)
	*Endomychobiusendomychi* (Walker, 1836)		
	*Epicopteruschoreiformis* Westwood, 1833		
	*Erdoesiatessellata* Bouček, 1957	CL01: *Erdoesiatesselata*; misspelliing	
	*Erdoesinaalboannulata* (Ratzeburg, 1852)		
	*Erythromalusrufiventris* (Walker, 1835)		Haas et al. (2021)
	*Eulonchetrontorymoides* (Thomson, 1878)		
	*Eumacepoluseinersbergensis* Ratzeburg, 1844	CL01: *Anogmuseinersbergensis* Ratzeburg	
	*Eumacepolussaxeseni* Graham, 1957		
	*Euneuralachni* (Ashmead, 1887)		
	*Euneurasaetosa* (Delucchi, 1955)		
	*Euneurasopolis* (Walker, 1844)		
	*Eunotusacutus* Kurdjumov, 1912		Hoffmann (2002)
	*Eunotusareolatus* (Ratzeburg, 1852)		
	*Eunotuscretaceus* Walker, 1834		Hoffmann (2002)
	*Eunotusnigriclavis* (Förster, 1856)		
	*Eunotusobscurus* Masi, 1931		
	*Eunotusparvulus* Masi, 1931		
	*Eurydinotaleptomera* Förster, 1878		
	*Gastracanthuspulcherrimus* Westwood, 1833		
	*Gastrancistrusacontes* Walker, 1840		
	*Gastrancistrusacutus* Graham, 1969		Haas et al. (2021)
	*Gastrancistrusaffinis* Graham, 1969		Haas et al. (2021)
	*Gastrancistrusamabilis* (Girault & Dodd, 1915)		
	*Gastrancistrusautumnalis* (Walker, 1834)		
	*Gastrancistrusclaviger* Förster, 1861		
	*Gastrancistruscompressus* Walker, 1834		Haas et al. (2021)
	*Gastrancistrusfulvicornis* (Walker, 1874)		
	*Gastrancistrusfulvicoxis* Graham, 1969		
	*Gastrancistrusfumipennis* Walker, 1834		Haas et al. (2021)
	*Gastrancistrusfuscicornis* Walker, 1834		
	*Gastrancistrusglabellus* (Nees, 1834)		
	*Gastrancistruslatifrons* (Thomson, 1876)		
	*Gastrancistruspicipes* (Nees, 1834)		
	*Gastrancistruspusztensis* (Erdös, 1946)		
	*Gastrancistrussalicis* (Nees, 1834)		
	*Gastrancistrustorymiformis* (Ratzeburg, 1852)		Wall (1973)
	*Gbelciacrassiceps* Bouček, 1961		Haas et al. (2021)
	*Glyphognathusconvexus* (Delucchi, 1953)		
	*Glyphognathusflammeus* (Delucchi, 1953)		
	*Glyphognathuslaevigatus* (Delucchi, 1953)		
	*Glyphognathusnitidus* (Delucchi, 1955)	CL01: *Sphaeripalpusnitidus* Delucchi	
	*Gyrinophagusaper* (Walker, 1839)		
	*Gyrinophagusluteipes* Ruschka, 1914		
	*Habritysbrevicornis* (Ratzeburg, 1844)		
	*Halticopteraaenea* (Walker, 1833)		
	*Halticopteracirculus* (Walker, 1833)		
	*Halticopteracollaris* (Walker, 1836)		
	*Halticopteracrius* (Walker1839)		
	*Halticopteradimidiata* (Förster, 1841)		
	*Halticopteraelongatula* Graham, 1972		
	*Halticopteraflavicornis* (Spinola, 1808)	CL01: *Halticopterasmaragdina* Curtis	
	*Halticopteraizzetbaysali* Doganlar, 2006		
	*Halticopteralaevigata* Thomson, 1876		
	*Halticopteralongipetiolus* Hedqvist, 1975		Haas et al. (2021)
	*Halticopteramustela* (Walker, 1839)		
	*Halticopterapatellana* (Dalman, 1818)		
	*Halticopterapolita* (Walker, 1834)		
	*Halticopterasmaragdina* (Walker, 1862)		
	*Halticopteratriannulata* (Erdös, 1946)		
	*Halticopteravehbikoci* Doganlar, 2006		
	*Hemitrichusseniculus* (Nees, 1834)		H. Baur det., von Heyden (1887)
	*Heteroprymnalongicornis* (Walker, 1835)		Haas et al. (2021)
	*Heydeniapretiosa* Förster, 1856		
	*Hobbyastenonota* (Ratzeburg, 1848)		
	*Holcaeuscalligetus* (Walker, 1839)		
	*Holcaeuscompressus* (Walker, 1836)		
	*Holcaeusdecipiens* (Thomson, 1878)		
	*Holcaeusgorgasus* (Walker, 1839)		
	*Holcaeusgracilentus* (Bouček, 1954)		
	*Holcaeusstenogaster* (Walker, 1836)		
	*Holcaeusstylatus* Graham, 1969		
	*Homoporusapharetus* (Walker, 1839)		Anton et al. (2020)
	*Homoporusarestor* (Walker, 1848)		
	*Homoporusdestructor* (Say, 1817)		Dorow et al. (1992)
	*Homoporusfebriculosus* (Girault, 1917)		
	*Homoporusfulviventris* (Walker, 1835)		Anton et al. (2020)
	*Homoporusgibbiscuta* (Thomson, 1878)		
	*Homoporusluniger* (Nees, 1834)		Dorow et al. (1992), Neugebauer (1997)
	*Homoporusnypsius* (Walker, 1839)		
	*Homoporuspulchripes* Erdös, 1953		Haas et al. (2021)
	*Homoporussemiluteus* (Walker, 1872)		
	*Homoporussubniger* (Walker, 1835)		Anton et al. (2020)
	*Hyperimeruspusillus* (Walker, 1833)		
	*Isocyrtuslaetus* Walker, 1833		
	*Janssoniellaambigua* Graham, 1969		
	*Janssoniellacaudata* Kerrich, 1957		
	*Kalevacorynocera* Graham, 1957		Haas et al. (2021)
	*Ksenoplataquadrata* Bouček, 1965		Haas et al. (2021)
	*Lampotermabianellatum* Graham, 1969		
	*Lampotermaviride* (Thomson, 1876)		
	*Lamprotatusannularis* (Walker, 1833)		
	*Lamprotatusbrevicornis* Thomson, 1876		
	*Lamprotatusclaviger* Thomson, 1876		
	*Lamprotatuspicinervis* Thomson, 1876		
	*Lamprotatuspschorni* Delucchi, 1953		
	*Lamprotatussimillimus* Delucchi, 1953		
	*Lamprotatussplendens* Westwood, 1833	CL01: *Lamprotatusornatus* Delucchi	
	*Lamprotatustruncatus* (Fonscolombe, 1832)	CL01: *Lamprotatuscerebrosus* Delucchi; *Lamprotatusmirabilis* Delucchi	
	*Lariophagusdistinguendus* (Förster, 1841)		
	*Lariophaguspuncticollis* (Möller, 1882)		
	*Lariophagusrufipes* Hedqvist, 1978		
	*Leptomeraporusnicaee* (Walker, 1839)		
	*Macroglenesbouceki* (Graham, 1969)		
	*Macrogleneschalybeus* (Haliday, 1833)		
	*Macroglenescompressus* (Förster, 1841)	CL01: *Stenophruscompressus* Förster	
	*Macroglenesconjungens* (Graham, 1969)		
	*Macrogleneseximius* (Haliday, 1833)		Haas et al. (2021)
	*Macroglenesgramineus* (Haliday, 1833)		Anton et al. (2020)
	*Macroglenesmicrocerus* Haliday, 1844		
	*Macroglenespaludum* (Graham, 1969)		Haas et al. (2021)
	*Macroglenespenetrans* (Kirby, 1800)		Anton et al. (2020)
	*Macroglenesvaricornis* (Haliday, 1833)		
	*Macromesusamphiretus* (Walker, 1848)		
	*Meraporusglaber* Szelényi, 1981	CL01: *Anisopteromalusglaber* Szelényi	
	*Meraporusgraminicola* (Walker, 1834)		
	*Merismusmegapterus* Walker, 1833		
	*Merismusnitidus* (Walker, 1833)		
	*Merismusrufipes* Walker, 1833		
	*Merismussplendens* Graham, 1969		
	*Merismusviridis* (Delucchi, 1955)		
	*Merisusflagellatus* Bouček, 1965		Dorow et al. (1992)
	*Merisussplendidus* Walker, 1834		Anton et al. (2020)
	*Mesopolobusaequus* (Walker, 1834)		
	*Mesopolobusalbitarsus* (Walker, 1834)		
	*Mesopolobusamaenus* (Walker, 1834)		
	*Mesopolobuscitrinus* (Ratzeburg, 1852)		
	*Mesopolobusclavicornis* (Förster, 1878)		
	*Mesopolobusdiffinis* (Walker, 1834)		
	*Mesopolobusdubius* (Walker, 1834)		
	*Mesopolobusfagi* Askew & Lampe, 1998		
	*Mesopolobusfasciiventris* Westwood, 1833		
	*Mesopolobusfuscipes* (Walker, 1834)		
	*Mesopolobusgemellus* Baur & Muller, 2007		
	*Mesopolobusgraminum* (Hardh, 1950)		Anton et al. (2020)
	*Mesopolobusincultus* (Walker, 1834)		
	*Mesopolobuslaticornis* (Walker, 1834)		
	*Mesopolobuslongicollis* Graham, 1969		
	*Mesopolobusmaculicornis* (Giraud, 1863)		
	*Mesopolobusmediterraneus* (Mayr, 1903)		
	*Mesopolobusmorys* (Walker, 1848)		Greiler (1993)
	*Mesopolobusnobilis* (Walker, 1834)		Anton et al. (2020)
	*Mesopolobusphragmitis* (Erdös, 1957)		
	*Mesopolobusprasinus* (Walker, 1834)		
	*Mesopolobusrhabdophagae* (Graham, 1957)		
	*Mesopolobussemiclavatus* (Ratzeburg, 1848)		
	*Mesopolobussericeus* (Forster, 1770)		
	*Mesopolobusspermotrophus* Hussey, 1960		
	*Mesopolobussubfumatus* (Ratzeburg, 1852)		H. Baur det.
	*Mesopolobustarsatus* (Nees, 1834)		
	*Mesopolobusteliformis* (Walker, 1834)		
	*Mesopolobustibialis* (Westwood, 1833)		
	*Mesopolobustypographi* (Ruschka, 1924)		
	*Mesopolobusverditer* (Norton, 1869)		
	*Mesopolobusxanthocerus* (Thomson, 1878)		
	*Metacolusazureus* (Ratzeburg, 1844)		
	*Metacolusunifasciatus* Förster, 1856		
	*Metastenusconcinnus* Walker, 1834		
	*Micradelusacutus* Graham, 1969		Haas et al. (2021)
	*Micradelusrotundus* Walker, 1834		
	*Miscogasterelegans* Walker, 1834		
	*Miscogasterhortensis* Walker, 1833		
	*Miscogastermaculata* Walker, 1833		
	*Miscogasternecopina* Delucchi, 1953		
	*Miscogasterrufipes* Walker, 1833	CL01: *Miscogasterfulgens* Delucchi	
	*Mokrzeckiapini* (Hartig, 1838)		von Heyden (1887)
	*Muscidifuraxraptor* Girault & Sanders, 1910		
	*Nasoniavitripennis* (Walker, 1836)		Franz and Wellenstein (1958), Greiler (1993), H. Baur det.
	*Nodisoplatadiffinis* (Walker, 1874)		
	*Norbanusobscurus* (Masi, 1922)		
	*Norbanusscabriculus* (Nees, 1834)		
	*Notanisussexramosus* (Erdös, 1946)		
	*Ooderaformosa* (Giraud 1863)		Werner and Peters (2018), Doczkal (2017)
	*Ormoceruslatus* Walker, 1834		
	*Ormocerusvernalis* Walker, 1834		
	*Oxyglyptarugosa* Ruschka, 1912		
	*Oxysychuspilosulus* (Thomson, 1878)		
	*Pachycrepoideusvindemmiae* (Rondani, 1875)		
	*Pachyneuronaphidis* (Bouché, 1834)		Blüthgen (1929)
	*Pachyneuronformosum* Walker, 1833		Anton et al. (2020)
	*Pachyneurongibbiscuta* Thomson, 1878		Thies et al. (1997)
	*Pachyneurongrande* Thomson, 1878		
	*Pachyneurongroenlandicum* (Holmgren, 1872)		
	*Pachyneuronleucopiscida* Mani, 1939		
	*Pachyneuronmuscarum* (Linnaeus, 1758)		Greiler (1993), Hoffmann (2002)
	*Pachyneuronnelsoni* Girault, 1928		
	*Pachyneuronplaniscuta* Thomson, 1878		
	*Pachyneuronsolitarium* (Hartig, 1838)		Blüthgen (1929)
	*Pachyneuronvitodurense* Delucchi, 1955		
	*Pandelusflavipes* (Förster, 1841)		
	*Panstenonagylla* (Walker, 1850)		
	*Panstenonoxylus* (Walker, 1839)		
	*Pegopusinornatus* (Walker, 1834)		
	*Peridesmiacongrua* (Walker, 1835)		
	*Peridesmiadiscus* (Walker, 1835)		
	*Perniphorarobusta* Ruschka, 1923		
	*Phaenocytusglechomae* (Förster, 1841)		
	*Platygerrhusaffinis* (Walker, 1836)		
	*Platygerrhusdolosus* (Walker, 1836)		
	*Platygerrhusductilis* (Walker, 1836)		
	*Platygerrhusunicolor* Graham, 1969		Haas et al. (2021)
	*Plutothrixbicolorata* (Spinola, 1808)		von Heyden (1887)
	*Plutothrixcoelius* (Walker, 1839)		
	*Plutothrixtrifasciata* (Thomson, 1878)		von Heyden (1887)
	*Pseudocatolaccusnitescens* (Walker, 1834)		
	*Psiloceraconfusa* Graham, 1992		Haas et al. (2021)
	*Psiloceracrassispina* (Thomson, 1878)		Haas et al. (2021)
	*Psiloceraobscura* Walker, 1833		
	*Psilocerapunctifrons* (Thomson, 1878)		
	*Psilonotusachaeus* Walker, 1848		
	*Psilonotusadamas* Walker, 1834		
	*Psilonotushortensia* Walker, 1846		
	*Psychophagoidescrassicornis* Graham, 1969		Haas et al. (2021)
	*Psychophagusomnivorus* (Walker, 1835)		
	*Pteromalusalbipennis* Walker, 1835		Nitschke et al. (2017)
	*Pteromalusaltus* (Walker, 1834)		Haas et al. (2021)
	*Pteromalusaureolus* (Thomson, 1878)		
	*Pteromalusbedeguaris* (Thomson, 1878)		Cölln (1998)
	*Pteromalusberylli* Walker, 1835		
	*Pteromalusbifoveolatus* Förster, 1861		
	*Pteromaluscardui* (Erdös, 1953)		Nitschke et al. (2017)
	*Pteromaluscaudiger* (Graham, 1969)		
	*Pteromaluschlorogaster* (Thomson, 1878)		
	*Pteromaluschlorospilus* (Walker, 1834)		
	*Pteromaluschrysos* Walker, 1836		Bachmeier (1965), Greiler (1993)
	*Pteromaluscioni* (Thomson, 1878)		
	*Pteromaluscionobius* (Erdös, 1953)		
	*Pteromalusconformis* (Graham, 1969)		
	*Pteromalusconopidarum* (Bouček, 1961)		
	*Pteromaluscrassinervis* (Thomson, 1878)		
	*Pteromaluscyniphidis* (Linnaeus, 1758)	CL01: *Pteromaluscapreae* Linnaeus	
	*Pteromalusdispar* (Curtis, 1827)		
	*Pteromalusdolichurus* (Thomson, 1878)		
	*Pteromaluselevatus* (Walker, 1834)		Nitschke et al. (2017)
	*Pteromaluseuurae* Askew, 1995		
	*Pteromalusfasciatus* (Thomson, 1878)		
	*Pteromalushieracii* (Thomson, 1878)		
	*Pteromalusintermedius* (Walker, 1834)		
	*Pteromaluslaricinellae* Ratzeburg, 1849		
	*Pteromalusmicrops* (Graham, 1969)		
	*Pteromalusmusaeus* Walker, 1844		
	*Pteromalusochrocerus* (Thomson, 1878)		Bathon and Ruppert (1996)
	*Pteromaluspapaveris* Förster, 1841		
	*Pteromalusparietinae* (Graham, 1969)		
	*Pteromaluspatro* Walker, 1848		
	*Pteromalusplatyphilus* Walker, 1874		
	*Pteromaluspuparum* (Linnaeus, 1758)		Greiler (1993), von Heyden (1887)
	*Pteromalusscandiae* (Graham, 1969)		
	*Pteromalussemotus* (Walker, 1834)		Greiler (1993)
	*Pteromalussequester* Walker, 1835		Kruess (1996)
	*Pteromalustereus* Walker, 1839		
	*Pteromalustripolii* Graham, 1969		
	*Pteromalusvarians* (Spinola, 1808)		
	*Pteromalusvibulenus* (Walker, 1839)		
	*Rakosinadeplanata* Bouček, 1956		
	*Rhaphitelusladenbergii* (Ratzeburg, 1844)		
	*Rhaphitelusmaculatus* Walker, 1834		
	*Rhicnocoeliaconstans* (Walker, 1836)		
	*Rhicnocoeliaimpar* (Walker, 1836)		Haas et al. (2021)
	*Rhopalicusguttatus* (Ratzeburg, 1844)		
	*Rhopalicusquadratus* (Ratzeburg, 1844)		Höller (1988)
	*Rhopalicustutela* (Walker, 1836)		von Heyden (1887)
	*Rohatinainermis* Bouček, 1954		Haas et al. (2021)
	*Rohatinamonstrosa* Bouček, 1954		Haas et al. (2021)
	*Roptrocerusmirus* (Walker, 1834)		
	*Roptrocerusxylophagorum* (Ratzeburg, 1844)		
	*Sceptrothelysdeione* (Walker, 1839)		
	*Sceptrothelysgrandiclava* (Walker, 1835)		
	*Sceptrothelysintermedia* Graham, 1969		
	*Sceptrothelysoccultus* (Förster, 1841)		
	*Schimitschekiapopuli* Bouček, 1965		Greiler (1993)
	*Schizonotuslatus* (Walker, 1835)		
	*Schizonotussieboldi* (Ratzeburg, 1848)		Georgi et al. (2012)
	*Seladermaberani* (Delucchi, 1953)		
	*Seladermabicolor* Walker, 1834	CL01: *Seladermaluteolum* Delucchi	
	*Seladermabreve* Walker, 1834		
	*Seladermacoeruleovirens* (Förster, 1861)		
	*Seladermaconvexum* Walker, 1834	CL01: *Seladermaagreste* Delucchi	
	*Seladermadiffine* (Walker, 1833)		
	*Seladermadiutinum* (Delucchi, 1955)		
	*Seladermageniculatum* (Zetterstedt, 1838)		
	*Seladermaglobosum* (Delucchi, 1953)		
	*Seladermalaetum* Walker, 1834	CL01: *Seladermanobile* Delucchi; *S.violaceum* Delucchi	
	*Seladermascaea* (Walker, 1844)		
	*Seladermasimplex* (Thomson, 1876)		
	*Semiotellusdiversus* (Walker, 1834)	CL01: *Seladermadiversus* Walker	
	*Semiotellusmundus* (Walker, 1834)		
	*Semiotellusrujanensis* Bouček, 1972		
	*Spalangiacameroni* Perkins, 1910		
	*Spalangiacrassicornis* Bouček, 1963		von Heyden (1887)
	*Spalangiaendius* Walker, 1839		
	*Spalangiaerythromera* Förster, 1850		
	*Spalangiafuscipes* Nees, 1834		
	*Spalangianigra* Latreille, 1805		
	*Spalangianigripes* Curtis, 1839		von Heyden (1887)
	*Spalangianigroaenea* Curtis, 1839		von Heyden (1887)
	*Spalangiarugulosa* Förster, 1850		
	*Spalangiasubpunctata* Förster, 1850		Anton et al. (2020)
	*Spalangiopeltaalata* Bouček, 1953		
	*Spalangiopeltadudichi* Erdös, 1955		Haas et al. (2021)
	*Spaniopusamoenus* Förster, 1856		
	*Spaniopusdissimilis* Walker, 1833		von Heyden (1887)
	*Spaniopuspeisonis* (Erdös, 1957)		
	*Sphaeripalpusfuscipes* (Walker, 1833)		
	*Sphaeripalpusviridis* Förster, 1841		
	*Sphegigastercuscutae* Ferrière, 1959		
	*Sphegigasterintersita* Graham, 1969		
	*Sphegigasternigricornis* (Nees, 1834)		
	*Sphegigasterpallicornis* (Spinola, 1808)		Neiber (2010)
	*Sphegigasterpedunculiventris* (Spinola, 1808)		
	*Sphegigastertruncata* (Thomson, 1878)		
	*Spintherusdubius* (Nees, 1834)		Kruess (1996)
	*Staurothyreuscruciger* Graham, 1956		
	*Stenomalinacommunis* (Nees, 1834)		
	*Stenomalinadives* (Walker, 1835)		
	*Stenomalinaepistena* (Walker, 1835)		
	*Stenomalinafavorinus* (Walker, 1839)		
	*Stenomalinagracilis* (Walker, 1834)		Kruess (1996), Anton et al. (2020)
	*Stenomalinaiera* (Walker, 1844)		
	*Stenomalinailludens* (Walker, 1836)		
	*Stenomalinalaticeps* (Walker, 1850)		
	*Stenomalinaliparae* (Giraud, 1863)		Schlemmermeyer (1994)
	*Stenomalinamicans* (Olivier, 1813)		
	Stenomalina oxygyne (Walker, 1835)		
	*Stenoselmanigrum* Delucchi, 1956	CL01: *Stenoselmanigrinum*; misspelling	
	*Stichocrepisarmata* (Förster, 1860)		Haas et al. (2021)
	*Stictomischusgibbus* (Walker, 1833)		
	*Stictomischusgroschkei* Delucchi, 1953		
	*Stictomischuslongiventris* Thomson, 1876		
	*Stictomischusminiatus* Delucchi, 1953		
	*Stictomischusnitentis* Delucchi, 1955		
	*Stictomischusobscurus* (Walker, 1833)		
	*Stictomischusscaposus* Thomson, 1876		
	*Stictomischustumidus* (Walker, 1833)		
	*Stinopluslapsanae* Graham, 1969		
	*Syntomopusincisus* Thomson, 1878		Lübbe (1990)
	*Syntomopusincurvus* Walker, 1833		Lübbe (1990)
	*Syntomopusoviceps* Thomson, 1878		Lübbe (1990)
	*Syntomopusthoracicus* Walker, 1833		
	*Systasisannulipes* (Walker, 1834)		Anton et al. (2020), Haas et al. (2021)
	*Systasisencyrtoides* Walker, 1834		von Heyden (1887)
	*Systasistenuicornis* Walker, 1834		
	*Termolampapinicola* Bouček, 1961		
	*Theocolaxelegans* (Westwood, 1874)		
	*Theocolaxformiciformis* Westwood, 1832		
	*Thinodytescyzicus* (Walker, 1839)		
	*Tomicobiapityophthori* (Bouček, 1955)		
	*Tomicobiaseitneri* (Ruschka, 1924)		
	*Toxeumaacilius* (Walker, 1848)		
	*Toxeumadiscretum* Graham, 1984		Haas et al. (2021)
	*Toxeumafuscicorne* Walker, 1833		
	*Trichomalusgermanus* (Dalla, Torre, 1889)	In Graham 1969 possible syn. of *Trichomalusconifer* (Walker)	Wall (1973)
	*Trichomalusgracilicornis* (Zetterstedt, 1838)		
	*Trichomalopsisacuminata* (Graham, 1969)	CL01: *Trichomalopsisacuminatus* Graham	
	*Trichomalopsisexigua* (Walker, 1834)		
	*Trichomalopsisfucicola* (Walker, 1835)		
	*Trichomalopsisgermanica* (Graham, 1969)		
	*Trichomalopsishemiptera* (Walker, 1835)		von Heyden (1887)
	*Trichomalopsismicroptera* (Lindeman, 1887)		
	*Trichomalopsisperegrina* (Graham, 1969)		
	*Trichomalopsispotatoriae* (Graham, 1969)		
	*Trichomalopsistigasis* (Walker, 1839)		
	*Trichomalusannulatus* (Förster, 1841)		
	*Trichomalusapertus* (Walker, 1835)		
	*Trichomalusbracteatus* (Walker, 1835)	CL01: *Trichomalusfasciatus* Förster	
	*Trichomaluscampestris* (Walker, 1834)		Kruess (1996)
	*Trichomalusconifer* (Walker, 1836)		
	*Trichomaluscoryphe* (Walker, 1839)		
	*Trichomaluselongatus* Delucchi & Graham, 1956		
	*Trichomalusflagellaris* Graham, 1969		
	*Trichomalusfrontalis* (Thomson, 1878)		
	*Trichomalusfulvipes* (Walker, 1836)		Kruess (1996)
	*Trichomalusgynetelus* (Walker, 1835)		
	*Trichomalushelvipes* (Walker, 1834)		Kruess (1996)
	*Trichomalusinscitus* (Walker, 1835)		Aerts (1955)
	*Trichomaluslepidus* (Förster, 1841)		
	*Trichomaluslonchaeae* Bouček, 1959		
	*Trichomaluslucidus* (Walker, 1835)		
	*Trichomalusnanus* (Walker, 1836)		
	*Trichomalusperfectus* (Walker, 1835)		Thies et al. (1997)
	*Trichomaluspexatus* (Walker, 1835)		
	*Trichomalusposticus* (Walker, 1834)		
	*Trichomalusrepandus* (Walker, 1835)		Kruess (1996)
	*Trichomalusrobustus* (Walker, 1835)		
	*Trichomalusrufinus* (Walker, 1835)		Anton et al. (2020)
	*Trichomalusrugosus* Delucchi & Graham, 1956		
	*Trichomalusstatutus* (Förster, 1841)		
	*Trichomalustenellus* (Walker, 1834)		
	*Tricolasxylocleptis* Bouček, 1967		
	*Tricyclomischuscelticus* Graham, 1956		Haas et al. (2021)
	*Trigonoderuscyanescens* (Förster, 1841)		Anton et al. (2020)
	*Trigonoderusfilatus* Walker, 1836		
	*Trigonoderusnobilitatus* Graham, 1993		Haas et al. (2021)
	*Trigonoderusprinceps* Westwood, 1832		
	*Tritneptisaffinis* (Nees, 1834)		
	*Tritneptisdiprionis* Gahan, 1938		
	*Tritneptisklugii* (Ratzeburg, 1844)		
	*Trychnosomapunctipleura* (Thomson, 1878)		Haas et al. (2021)
	*Urolepismaritima* (Walker, 1834)		
	*Urolepisrufipes* (Ashmead, 1896)		
	*Vrestoviafidenas* (Walker, 1848)		
	*Xestomnasterchrysochlorus* (Walker, 1846)		
	*Xestomnastermazares* (Walker, 1844)		
	*Xiphydriophagusmeyerinckii* (Ratzeburg, 1848)		
	*Yusufiaacerina* (Bouček, 1972)		
**Signiphoridae**			
	*Chartocerussubaeneus* (Förster, 1878)		
	*Thysanusater* Walker, 1840		Anton et al. (2020)
**Tetracampidae**			
	*Dipriocampediprioni* (Ferrière, 1935)		
	*Epiclerusnomocerus* (Masi, 1934)		Neiber (2010)
	*Epicleruspanyas* (Walker, 1839)		
	*Epiclerustemenus* (Walker, 1839)		
	*Foersterellaerdoesi* Bouček, 1958		
	*Foersterellafuscicornis* Hansson, 2016		
	*Foersterellareptans* (Nees, 1834)		
	*Platynocheiluscuprifrons* (Nees, 1834)		
	*Tetracampeimpressa* Förster, 1841		
**Torymidae**			
	*Cryptopristuscaliginosus* (Walker, 1833)	CL01: *Eriodontomerusilliesi* Szelényi	
	*Eridontomerusisosomatis* (Riley, 1882)		
	*Eridontomeruslaticornis* (Förster, 1859)		
	*Eridontomerussyrphi* (Förster, 1859)		
	*Exopristustrigonomerus* (Masi, 1916)		
	*Glyphomerusstigma* (Fabricius, 1793)		Cölln (1998)
	*Glyphomerustibialis* Förster, 1856		
	*Idiomacromeruspapaveris* (Förster, 1856)		
	*Idiomacromerusterebrator* (Masi, 1916)	CL01: *Idiomacromeruslongfellowi* Girault	
	*Microdontomerusannulatus* (Spinola, 1808)		Nitschke et al. (2017)
	*Monodontomerusaeneus* (Fonscolombe, 1832)		
	*Monodontomerusaereus* Walker, 1834		
	*Monodontomerusdentipes* (Dalman, 1820)		von der Dunk (1987)
	*Monodontomeruslaricis* Mayr, 1874		
	*Monodontomerusminor* (Ratzeburg, 1848)		
	*Monodontomerusobscurus* Westwood, 1833		Anton et al. (2020)
	*Monodontomerusvicicellae* (Walker, 1847)		
	*Podagrionpachymerum* (Walker, 1833)		Petrischak and Ulrich (2012)
	*Pseudotorymusarvernicus* (Walker, 1833)	CL01: *Pseudotorymusdubius* Nees	Kruess (1996)
	*Pseudotorymusleguminus* Ruschka, 1923		
	*Pseudotorymusmedicaginis* (Mayr, 1874)		Anton et al. (2020)
	*Pseudotorymusmilitaris* (Boheman, 1834)		
	*Pseudotorymusnapi* (Amerling & Kirchner, 1860)		
	*Pseudotorymuspapaveris* (Thomson, 1876)		
	*Pseudotorymussalicis* Ruschka, 1923		
	*Pseudotorymussalviae* Ruschka, 1923		
	*Pseudotorymussapphyrinus* (Fonscolombe, 1832)		
	*Torymoideskiesenwetteri* (Mayr, 1874)		Nitschke et al. (2017)
	*Torymusaffinis* (Fonscolombe, 1832)		
	*Torymusarcticus* (Thomson, 1876)		Claessens et al. (2016)
	*Torymusargei* Bouček, 1994		
	*Torymusarmatus* (Boheman, 1834)		
	*Torymusartemisiae* Mayr, 1874		
	*Torymusarundinis* (Walker, 1833)		Schlemmermeyer (1994)
	*Torymusauratus* (Müller, 1764)		
	*Torymusaustriacus* Graham, 1994		
	*Torymusazureus* Boheman, 1834		
	*Torymusbasalis* (Walker, 1833)		
	*Torymusbaudysi* Bouček, 1954		
	*Torymusbedeguaris* (Linnaeus, 1758)		
	*Torymuscalcaratus* (Nees, 1834)		
	*Torymuscaudatus* Boheman, 1834		
	*Torymuscerri* (Mayr, 1874)		
	*Torymuschloromerus* (Walker, 1833)		Nitschke et al. (2017)
	*Torymuschrysocephalus* Boheman, 1834		
	*Torymuscingulatus* Nees, 1834		
	*Torymusconfinis* (Walker, 1833)		Lübbe (1990)
	*Torymuscultriventris* Ratzeburg, 1844		
	*Torymuscupreus* (Spinola, 1808)		
	*Torymuscyaneus* Walker, 1847		
	*Torymusdruparum* Boheman, 1834		Anton et al. (2020)
	*Torymuseadyi* Graham & Gijswijt, 1998		
	*Torymuseglanteriae* Mayr, 1874	CL01: *Torymuseleganteriae*; misspelling	
	*Torymusepilobii* Graham & Gijswijt, 1998		
	*Torymuserucarum* (Schrank, 1781)		
	*Torymusfagi* (Hoffmeyer, 1930)		
	*Torymusfagineus* Graham, 1994		
	*Torymusfastuosus* Boheman, 1834		
	*Torymusflavipes* (Walker, 1833)		
	*Torymusformosus* (Walker, 1833)		
	*Torymusfuscicornis* (Walker, 1833)		
	*Torymusgalii* Boheman, 1834		
	*Torymusgeranii* (Walker, 1833)		
	*Torymusheyeri* Wachtl, 1833		
	*Torymusigniceps* Mayr, 1874		
	*Torymusimpar* Rondani, 1877		
	*Torymuslaetus* (Walker, 1833)		
	*Torymuslongicalcar* Graham, 1994		
	*Torymusmicrocerus* (Walker, 1833)		
	*Torymusmicrostigma* (Walker, 1833)		
	*Torymusmicrurus* Bouček, 1994		
	*Torymusnitidulus* (Walker, 1833)		
	*Torymusnobilis* Boheman, 1834		
	*Torymusnotatus* (Walker, 1833)		
	*Torymuspascuorum* Bouček, 1994		
	*Torymuspastinacae* Graham & Gijswijt, 1998		
	*Torymuspersicariae* Mayr, 1874		
	*Torymusphillyreae* Ruschka, 1921		
	*Torymuspoae* (Hoffmeyer, 1930)		
	*Torymusquercinus* Boheman, 1834		
	*Torymusroboris* (Walker, 1833)		
	*Torymusrosariae* Graham & Gijswijt, 1998		
	*Torymusrubi* (Schrank, 1781)		
	*Torymusruschkai* (Hoffmeyer, 1930)		
	*Torymusscutellaris* (Walker, 1833)		
	*Torymusseminum* (Hoffmeyer, 1929)		
	*Torymussinensis* Kamijo, 1982		
	*Torymusspeciosus* Boheman, 1834		
	*Torymusspinosus* (Kamijo, 1979)		
	*Torymustanaceticola* Ruschka, 1921		
	*Torymustipulariarum* Zetterstedt, 1838		
	*Torymusvarians* (Walker, 1833)		
	*Torymusventralis* (Fonscolombe, 1832)		
**Trichogrammatidae**			
	*Asynactaexigua* (Nees, 1834)		
	*Chaetostrichawalkeri* (Förster, 1851)		
	*Chaetostrichellapungens* (Mayr, 1904)		
	*Lathromerisdanica* (Kryger, 1919)		
	*Lathromerisgermanica* (Girault, 1914)		
	*Lathromerisscutellaris* Förster, 1856		Anton et al. (2020)
	*Oligositafoersteri* Girault, 1914		Anton et al. (2020)
	*Oligositasubfasciata* Westwood, 1879		
	*Ophioneurussignatus* Ratzeburg, 1852		
	*Poropoeastollwerckii* Förster, 1851		
	*Prestwichiaaquatica* Lubbock, 1864		
	*Prestwichiasolitaria* Ruschka, 1913		
	*Pseudoligositanigripes* (Girault, 1914)		
	*Trichogrammabrassicae* Bezdenko, 1968		
	*Trichogrammacacaeciae* Marchal, 1927		
	*Trichogrammacephalciae* Hochmut & Martinek, 1963		
	*Trichogrammachilonis* Ishii, 1941		
	*Trichogrammadendrolimi* Matsumura, 1926		
	*Trichogrammaembryophagum* (Hartig, 1838)		
	*Trichogrammaeuproctidis* (Girualt, 1911)		
	*Trichogrammaevanescens* Westwood, 1833		
	*Trichogrammaminutum* Riley, 1871		
	*Trichogrammasemblidis* (Aurivillius, 1898)		
	*Trichogrammazeirapherae* Walter, 1985		
	*Trichogrammatoideastammeri* (Novicky, 1946)		
	*Ufensfoersteri* (Kryger, 1919)		
**Mymarommatidae (Mymarommatoidea)**			
	*Mymarommaanomalum* (Blood & Kryger, 1922)	CL01: *Paleomymaranomalum* (Blood & Kryger)	

**Table 3.** List of species excluded from the present checklist. The species were included in Vidal (2001) or erroneously omitted from the previous checklist by Vidal (2001). For details see information in the main text and comments given for each species.

**Family**	**Species**	**Comments**
Aphelinidae	*Aphelinuscircumscriptus* (Ratzeburg, 1852)	Not included in Ferrière (1965).
Aphelinidae	*Aphelinusflavus* (Nees, 1834)	Interpretation of the species is unclear.
Chalcididae	*Belaspidiaobscura* Masi, 1916	According to Delvare et al. (1999), the Central European specimens of *B.obscura* sensu Bouček belongs to *B.nigra* Masi; *B.obscura* is west-Mediterranean and not verified in Germany.
Chalcididae	*Chalcisramicornis* Gravenhorst, 1834	Doubtful species status.
Encyrtidae	*Encyrtusflavipes* Nees, 1834	Not listed in Trjapitzin (1989) for Germany.
Encyrtidae	*Encyrtusfoersteri* Mayr, 1876	Not listed in Trjapitzin (1989) for Germany.
Encyrtidae	*Encyrtusmucronatus* Ratzeburg, 1848	Not listed in Trjapitzin (1989) for Germany.
Encyrtidae	*Leptomastixhistrio* (Förster, 1856)	According to Trjapitzin (1989) uncertain collecting location.
Encyrtidae	*Zaommahirsuta* (Ratzeburg, 1852)	Trjapitzin (1989): name unclear, Graham (1969): possible synonym of *Apterencyrtusmicrophagus* (Mayr).
Eucharitidae	*Stilbulacyniformis* (Rossius, 1792)	Listed erronously in UCD
Eulophidae	*Aprostocetusflavovarius* (Nees, 1834)	Graham (1987): possible synonym of *A.forsteri* (Walker).
Eulophidae	*Aulogymnusfumatus* (Ratzeburg, 1852)	Doubtful species status.
Eulophidae	*Chrysocharisfoliincolarum* (Christ, 1791)	Not included in Hansson (1985).
Eulophidae	*Elachertusaequalis* Förster, 1841	Bouček and Askew (1968): incertae sedis.
Eulophidae	*Elachertusaeruginosus* Förster, 1841	Bouček and Askew (1968): incertae sedis.
Eulophidae	*Elachertusalbipes* Nees, 1841	Bouček and Askew (1968): incertae sedis; type probably lost.
Eulophidae	*Elachertusalbitarsus* (Ratzeburg, 1844)	Bouček and Askew (1968): incertae sedis; type probably lost.
Eulophidae	*Elachertuscoerulescens* Nees, 1834	Bouček and Askew (1968): incertae sedis; type probably lost.
Eulophidae	*Elachertuscyaneus* Förster, 1841	Bouček and Askew (1968): incertae sedis; type probably lost.
Eulophidae	*Elachertusdeplanatus* (Ratzeburg, 1852)	Bouček and Askew (1968): incertae sedis.
Eulophidae	*Elachertusditissimus* Förster, 1841	Bouček and Askew (1968): incertae sedis; type probably lost.
Eulophidae	*Elachertusfacialis* Förster, 1841	Bouček and Askew (1968): incertae sedis; type probably lost.
Eulophidae	*Elachertuslaevis* Förster, 1841	Bouček and Askew (1968): incertae sedis; doubtful species status.
Eulophidae	*Elachertuslunatus* Förster, 1841	Bouček and Askew (1968): incertae sedis.
Eulophidae	*Elachertusorbicularis* Nees, 1834	Bouček and Askew (1968): incertae sedis; type probably lost.
Eulophidae	*Elachertuspallipes* Nees, 1834	Bouček and Askew (1968): incertae sedis; type probably lost.
Eulophidae	*Elachertuspellucens* Förster, 1841	Bouček and Askew (1968): incertae sedis.
Eulophidae	*Elachertusplagiatus* Förster, 1841	Bouček and Askew (1968): incertae sedis; type probably lost.
Eulophidae	*Elachertuspulcherrimus* Förster, 1841	Bouček and Askew (1968): incertae sedis.
Eulophidae	*Elachertusreticulatus* Ratzeburg, 1852	Bouček and Askew (1968): incertae sedis; type probably lost.
Eulophidae	*Elachertussubauratus* Nees, 1834	Bouček and Askew (1968): incertae sedis; type probably lost.
Eulophidae	*Elachertustardulus* Nees, 1834	Bouček and Askew (1968): incertae sedis.
Eulophidae	*Elachertustimidus* Förster 1841	Doubtful species status.
Eulophidae	*Elachertuswalkeri* (Ratzeburg, 1848)	Bouček and Askew (1968): incertae sedis; type probably lost.
Eulophidae	*Entedonaequilongus* Ratzeburg, 1852	In Graham (1971) not listed for Germany.
Eulophidae	*Entedoncaudatus* Ratzeburg, 1848	Bouček and Askew (1968): incertae sedis; type probably lost.
Eulophidae	*Entedoncavicornis* Ratzeburg, 1852	Bouček and Askew (1968): incertae sedis; type probably lost; in Graham (1971) not listed for Germany.
Eulophidae	*Entedonchalybaeus* Ratzeburg, 1852	Bouček and Askew (1968): incertae sedis; type probably lost.
Eulophidae	*Entedonconfinis* Ratzeburg, 1848	Bouček and Askew (1968): incertae sedis; type probably lost; in Graham (1971) not listed for Germany.
Eulophidae	*Entedonconnexus* Ratzeburg, 1852	Bouček and Askew (1968): incertae sedis; type probably lost; in Graham (1971) not listed for Germany.
Eulophidae	*Entedondebilis* Ratzeburg, 1852	Bouček and Askew (1968): incertae sedis; type probably lost.
Eulophidae	*Entedonfumatus* Ratzeburg, 1852	Bouček and Askew (1968): incertae sedis; type probably lost.
Eulophidae	*Entedonhylotomarum* (Ratzeburg, 1844)	In Graham (1971) not listed for Germany.
Eulophidae	*Entedoninconspicuus* Ratzeburg, 1852	In Graham (1971) not listed for Germany.
Eulophidae	*Entedonlongiventris* Ratzeburg, 1848	Bouček and Askew (1968): incertae sedis; type probably lost.
Eulophidae	*Entedonluteipes* Ratzeburg, 1848	Bouček and Askew (1968): incertae sedis; type probably lost; in Graham (1971) not listed for Germany.
Eulophidae	*Entedonpinetorum* Ratzeburg, 1852	Graham (1991): incertae sedis.
Eulophidae	*Entedontransparens* Ratzeburg, 1848	Bouček and Askew (1968): incertae sedis; type probably lost; in Graham (1971) not listed for Germany.
Eulophidae	*Entedonunicostatus* Ratzeburg ,1848	Bouček and Askew (1968): incertae sedis; type probably lost; in Graham (1971) not listed for Germany.
Eulophidae	*Entedonvaginulae* Ratzeburg, 1852	In Graham (1971) not listed for Germany.
Eulophidae	*Entedonxanthostoma* (Ratzeburg, 1844)	In Graham (1971) not listed for Germany.
Eulophidae	*Eulophusater* Nees, 1834	Bouček and Askew (1968): incertae sedis; type probably lost.
Eulophidae	*Eulophusalbitarsus* Ratzeburg, 1844	In Bouček (1959) not listed for Germany.
Eulophidae	*Eulophusbinotatus* Förster, 1841	In Bouček (1959) not listed for Germany.
Eulophidae	*Eulophusblancardellae* Bouché, 1834	In Bouček (1959) not listed for Germany.
Eulophidae	*Eulophusbreviramulis* Förster, 1841	In Bouček (1959) not listed for Germany.
Eulophidae	*Eulophuscephalotes* Nees, 1834	Bouček and Askew (1968): incertae sedis; type probably lost; in Bouček (1959) not listed for Germany.
Eulophidae	*Eulophuschrysomelae* Nees, 1834	In Bouček (1959) not listed for Germany.
Eulophidae	*Eulophuscoccorum* Ratzeburg, 1848	Bouček and Askew (1968): incertae sedis; type probably lost; in Bouček (1959) not listed for Germany.
Eulophidae	*Eulophusdepressus* Nees, 1834	In Bouček (1959) not listed for Germany.
Eulophidae	*Eulophusdubitabilis* Förster, 1841	Bouček and Askew (1968): incertae sedis; type probably lost; in Bouček (1959) not listed for Germany.
Eulophidae	*Eulophusemicans* Nees, 1834	Bouček and Askew (1968): incertae sedis; type probably lost; in Bouček (1959) not listed for Germany.
Eulophidae	*Eulophuseurytomae* Nees, 1834	Bouček and Askew (1968): incertae sedis; type probably lost.
Eulophidae	*Eulophusfissicornis* Förster, 1841	Bouček and Askew (1968): incertae sedis; type probably lost.
Eulophidae	*Eulophusfoveolatus* Nees, 1834	Bouček and Askew (1968): incertae sedis; type probably lost; in Bouček (1959) not listed for Germany.
Eulophidae	*Eulophusfuscicornis* Förster, 1841	Bouček and Askew (1968): incertae sedis; type probably lost.
Eulophidae	*Eulophusharmocerus* Förster, 1841	Bouček and Askew (1968): incertae sedis.
Eulophus	*Eulophushylesinorum* Ratzeburg, 1844	Bouček and Askew (1968): incertae sedis; type probably lost.
Eulophidae	*Eulophusinconspicuus* Nees, 1834	Bouček and Askew (1968): incertae sedis; type probably lost; in Bouček (1959) not listed for Germany.
Eulophidae	*Eulophuslucens* Nees, 1834	Bouček and Askew (1968): incertae sedis; type probably lost.
Eulophidae	*Eulophusmedius* Nees, 1834	Bouček and Askew (1968): incertae sedis; type probably lost.
Eulophidae	*Eulophusmetallicus* Nees, 1834	Bouček and Askew (1968): incertae sedis; type probably lost.
Eulophidae	*Eulophusnitidulus* Nees, 1834	Bouček and Askew (1968): incertae sedis; type probably lost; in Bouček (1959) not listed for Germany.
Eulophidae	*Eulophusobscurus* Ratzeburg, 1848	Bouček and Askew (1968): incertae sedis; type probably lost.
Eulophidae	*Eulophuspolycerus* Förster, 1841	Bouček and Askew (1968): incertae sedis; type probably lost; in Bouček (1959) not listed for Germany.
Eulophidae	*Eulophusrupicapra* Förster, 1841	Bouček and Askew (1968): incertae sedis; type probably lost; in Bouček (1959) not listed for Germany.
Eulophiae	*Eulophusscutellaris* Nees, 1834	Bouček and Askew (1968): incertae sedis; type probably lost.
Eulophidae	*Eulophussemicupreus* Nees, 1834	Bouček and Askew (1968): incertae sedis; type probably lost; in Bouček (1959) not listed for Germany.
Eulophidae	*Eulophussexradiatus* Förster,1841	Bouček and Askew (1968): incertae sedis.
Eulophidae	*Eulophustabidus* Nees, 1834	Bouček and Askew (1968): incertae sedis; type probably lost; in Bouček (1959) not listed for Germany.
Eulophidae	*Eulophustarandus* Förster, 1841	Bouček and Askew (1968): incertae sedis.
Eulophidae	*Eulophustibialis* Nees, 1834	Bouček and Askew (1968): incertae sedis; type probably lost.
Eulophidae	*Eulophusvagus* Nees, 1834	Bouček and Askew (1968): incertae sedis; type probably lost; in Bouček (1959) not listed for Germany.
Eulophidae	*Eulophusxanthostoma* Ratzeburg, 1844	Bouček and Askew (1968): incertae sedis.
Eulophidae	*Pnigaliocruciatus* (Ratzeburg, 1848)	Bouček and Askew (1968): incertae sedis; type probably lost; Graham (1969): possible synonym of *P.agraules* Walker.
Eulophidae	*Pnigalioobscurus* (Ratzeburg, 1848)	Bouček and Askew (1968): incertae sedis.
Eulophidae	*Pnigaliotardulus* (Nees, 1834)	Graham (1969): possible synonym of *P.pectinicornis* L.
Eulophidae	*Tetrastichusatrocoeruleus* (Nees, 1834)	Not included in Hansson and Schmidt (2020).
Eulophidae	*Tetrastichuscapitatus* (Ratzeburg, 1852)	Not included in Hansson and Schmidt (2020).
Eulophidae	*Tetrastichusinunctus* (Ratzeburg, 1848)	Not included in Hansson and Schmidt (2020).
Eulophidae	*Tetrastichusooctonus* (Kawall, 1858)	Not included in Hansson and Schmidt (2020).
Eupelmidae	*Eupelmusbrachynterae* (Schwägrichen, 1835)	Gibson and Fusu (2016): name unclear.
Eupelmidae	*Eupelmushostilis* Förster, 1860	Gibson and Fusu (2016): Synonym of *E.urozonus* Dalman.
Eupelmidae	*Metapelmainsignis* (Förster, 1860)	Gibson and Fusu (2016): name unclear, the type (male) is an unidentifiable *Anastatus*; in Vidal (2001) as *M.insignis* (Förster).
Eurytomidae	*Eurytomaabrotani* (Panzer, 1801)	Not included in Zerova (1995).
Eurytomidae	*Eurytomaaterrima* (Schrank, 1781)	Not included in Zerova (1995).
Eurytomidae	*Eurytomaextincta* Ratzeburg, 1852	Not included in Zerova (1995).
Eurytomidae	*Eurytomagracilior* Dalla Torre, 1898	Not included in Zerova (1995).
Eurytomidae	*Eurytomamicroneura* Ratzeburg, 1852	Not included in Zerova (1995).
Eurytomidae	*Eurytomanodulosa* Ratzeburg, 1848	Not included in Zerova (1995).
Eurytomidae	*Eurytomapetiolata* Förster, 1841	Not included in Zerova (1995).
Eurytomidae	*Eurytomapumila* Förster, 1841	Not included in Zerova (1995).
Eurytomidae	*Eurytomapunctulata* Förster, 1841	Not included in Zerova (1995).
Eurytomidae	*Eurytomascabra* Förster, 1841	Not included in Zerova (1995).
Eurytomidae	*Eurytomasphegum* (Fabricius, 1787)	Not included in Zerova (1995).
Eurytomidae	*Tetramesaflavipes* (Förster, 1841)	Doubtful species status.
Megastigmidae	*Bootanomyiabohemanii* (Ratzeburg, 1848)	Doubtful species status.
Mymaridae	*Alaptusschmitzi* Soyka, 1939	Triapitsyn (2017): type location in Poland, not Gemany; in Triapitsyn et al. (2020) not listed for Germany.
Mymaridae	*Anaphesangustipennis* (Debauche, 1948)	In Huber and Thuróczy (2018) not listed for Germany, synonym of *A.ovipositor* Soyka.
Mymaridae	*Erythmelusfoersteri* (Ratzeburg, 1848)	Doubtful species status.
Ormyridae	*Ormyruschalybeus* (Ratzeburg, 1844)	Not included in Zerova and Seryogina (2006).
Ormyridae	*Ormyruscosmozonus* Förster, 1860	Förster (1860): described from Tyrol.
Ormyridae	*Ormyruspunctulatus* (Förster, 1860)	Not included in Zerova and Seryogina (2006).
Ormyridae	*Ormyrusversicolor* Förster, 1860	Not included in Zerova and Seryogina (2006).
Ormyridae	*Ormyrusviolaceus* Förster, 1860	Not included in Zerova and Seryogina (2006).
Perilampidae	*Chrysolampusaeneicornis* Ratzeburg, 1852	Not included in Graham (1969) and Doganlar and Doganlar (2014).
Perilampidae	*Chrysolampusanguliventris* Nees, 1834	Not included in Graham (1969) and Doganlar and Doganlar (2014).
Perilampidae	*Chrysolampusattenuatus* Förster, 1841	Not included in Graham (1969) and Doganlar and Doganlar (2014).
Perilampidae	*Chrysolampusbrevicornis* Förster, 1841	Not included in Graham (1969) and Doganlar and Doganlar (2014).
Perilampidae	*Chrysolampuscoeruleovirens* Förster, 1841	Not included in Graham (1969) and Doganlar and Doganlar (2014).
Perilampidae	*Chrysolampusdubius* Förster, 1841	Not included in Graham (1969) and Doganlar and Doganlar (2014).
Perilampidae	*Chrysolampusellipticus* Förster, 1841	Not included in Graham (1969) and Doganlar and Doganlar (2014).
Perilampidae	*Chrysolampusexcellens* Förster, 1841	Not included in Graham (1969) and Doganlar and Doganlar (2014).
Perilampidae	*Chrysolampusfuscimanus* Förster, 1841	Not included in Graham (1969) and Doganlar and Doganlar (2014).
Perilampidae	*Chrysolampusgibbosus* Förster, 1841	Not included in Graham (1969) and Doganlar and Doganlar (2014).
Perilampidae	*Chrysolampusgilvipes* Förster, 1841	Not included in Graham (1969) and Doganlar and Doganlar (2014).
Perilampidae	*Chrysolampusgranulatus* Förster, 1841	Not included in Graham (1969) and Doganlar and Doganlar (2014), in Vidal (2001) as *C.granularis* Förster.
Perilampidae	*Chrysolampusindubitatus* Förster, 1841	Not included in Graham (1969) and Doganlar and Doganlar (2014).
Perilampidae	*Chrysolampusinterruptus* Förster, 1841	Not included in Graham (1969) and Doganlar and Doganlar (2014).
Perilampidae	*Chrysolampuslaevipetiolatus* Förster, 1841	Not included in Graham (1969) and Doganlar and Doganlar (2014).
Perilampidae	*Chrysolampuspachymerus* Förster, 1841	Not included in Graham (1969) and Doganlar and Doganlar (2014).
Perilampidae	*Chrysolampuspallitarsis* Förster, 1841	Not included in Graham (1969) and Doganlar and Doganlar (2014).
Perilampidae	*Chrysolampusscapularis* Ratzeburg, 1852	Not included in Graham (1969) and Doganlar and Doganlar (2014).
Perilampidae	*Chrysolampussubcarinatus* Förster, 1841	Not included in Graham (1969) and Doganlar and Doganlar (2014).
Perilampidae	*Chrysolampussubsessilis* Nees, 1834	Not included in Graham (1969) and Doganlar and Doganlar (2014).
Perilampidae	*Chrysolampustenuiscapus* Förster, 1841	Not included in Graham (1969) and Doganlar and Doganlar (2014).
Perilampidae	*Chrysolampustransversus* Förster, 1841	Not included in Graham (1969) and Doganlar and Doganlar (2014).
Perilampidae	*Perilampusangustus* Nees, 1834	Not included in Bouček (1956).
Pteromalidae	*Acroclisisnigricornis* Förster, 1878	Not included in Graham (1969).
Pteromalidae	*Caenacisflavipes* Masi, 1931	Not included in Graham (1969); Masi (1911): specimens in HMB, collected by Strand in Norway and Germany, status unclear.
Pteromalidae	*Cleonymusapicalis* Förster, 1841	Not included in Graham (1969).
Pteromalidae	*Cleonymuscyaneus* Förster, 1841	Not included in Graham (1969).
Pteromalidae	*Cleonymuselongatus* Förster, 1841	Not included in Graham (1969).
Pteromalidae	*Cleonymuseximius* Förster, 1841	Not included in Graham (1969).
Pteromalidae	*Cleonymusviridinitens* Förster, 1841	Not included in Graham (1969).
Pteromalidae	*Coelopisthiaeurynota* (Förster, 1841)	Graham (1969): possible synonym of *Coelopisthiaextenta* (Walker).
Pteromalidae	*Dirhicnusclandestinus* (Förster, 1841)	Not included in Graham (1969).
Pteromalidae	*Gastracanthuserythrogaster* (Dalla Torre, 1898)	Not included in Graham (1969).
Pteromalidae	*Halticopteracorrusca* (Gravenhorst, 1807)	Not included in Graham (1969).
Pteromalidae	*Halticopteraplana* (Förster, 1841)	Not included in Graham (1969).
Pteromalidae	*Holcaeusglabriculus* (Nees, 1834)	Not included in Graham (1969).
Pteromalidae	*Holcaeussiccatorum* (Ratzeburg, 1852)	Not included in Graham (1969).
Pteromalidae	*Homoporusfemoralis* (Förster, 1841)	Not included in Graham (1969).
Pteromalidae	*Lariophagusteutonus* (Dalla Torre, 1898)	Doubtful species status.
Pteromalidae	*Macroglenesbrevicornis* (Nees, 1834)	Not included in Graham (1969), Mitroiu (2010): possible synonym of *M.chalybaeus* (Haliday); Dale-Skey et al. (2016): invalid.
Pteromalidae	*Meraporusfoveolatus* (Förster, 1841)	Not included in Graham (1969).
Pteromalidae	*Meraporusmodestus* (Förster, 1841)	Not included in Graham (1969).
Pteromalidae	*Mesopolobusbidentis* (Ratzeburg, 1848)	Not included in Graham (1969).
Pteromalidae	*Mesopolobusclavatus* (Ratzeburg, 1844)	Not included in Graham (1969).
Pteromalidae	*Mesopolobuscrassipes* (Ratzeburg, 1844)	Not included in Graham (1969).
Pteromalidae	*Mesopolobusdilutipes* (Ratzeburg, 1848)	Not included in Graham (1969).
Pteromalidae	*Metacolusazurescens* (Ratzeburg, 1852)	Graham (1969): possible synonym of *M.azureus* (Ratzeburg).
Pteromalidae	*Mokrzeckiahalidayana* (Ratzeburg, 1848)	Tselikh and Целих (2012): Synonym of *M.pini* (Hartig).
Pteromalidae	*Neocatolaccusproximus* (Förster, 1841)	Not included in Graham (1969).
Pteromalidae	*Pachyneuronflavipes* (Förster, 1841)	Not included in Graham (1969).
Pteromalidae	*Pachyneuroninnoxius* (Förster 1841)	Not included in Graham (1969).
Pteromalidae	*Pachyneuronpiceae* (Ratzeburg, 1848)	Not included in Graham (1969).
Pteromalidae	*Pachyneuronratzeburgi* Özdikmen, 2011	Not included in Graham (1969).
Pteromalidae	*Polycystusoscinidis* Kurdjumov, 1958	Noyes (2019): unavailable name, genus synonym of *Cyrtogaster* (Dale-Skey et al. 2016).
Pteromalidae	*Psiloceraverticillata* (Förster, 1841)	Not included in Graham (1969).
Pteromalidae	*Pteromalusaberrans* Förster, 1841	Not included in Graham (1969).
Pteromalidae	*Pteromalusabieticola* Ratzeburg, 1848	Not included in Graham (1969).
Pteromalidae	*Pteromalusacicularis* Förster, 1841	Not included in Graham (1969).
Pteromalidae	*Pteromalusacuminatus* Förster, 1841	Not included in Graham (1969).
Pteromalidae	*Pteromalusaequus* Förster, 1841	Not included in Graham (1969).
Pteromalidae	*Pteromalusaerosus* Förster, 1841	Not included in Graham (1969).
Pteromalidae	*Pteromalusagilis* Förster, 1841	Not included in Graham (1969).
Pteromalidae	*Pteromalusalbescens* Ratzeburg, 1844	Not included in Graham (1969).
Pteromalidae	*Pteromalusalternans* Förster, 1841	Not included in Graham (1969).
Pteromalidae	*Pteromalusambiguus* Förster, 1861	Not included in Graham (1969).
Pteromalidae	*Pteromalusangustus* Förster, 1841	Not included in Graham (1969).
Pteromalidae	*Pteromalusanomalipennis* Förster, 1841	Doubtful species status, in Vidal (2001) as *P.anomalipes* Förster.
Pteromalidae	*Pteromalusapicalis* Nees, 1834	Doubtful species status.
Pteromalidae	*Pteromalusarborivagus* Förster, 1841	Doubtful species status.
Pteromalidae	*Pteromalusatramentarius* Förster, 1841	Doubtful species status.
Pteromalidae	*Pteromalusaurantiacus* (Ratzeburg, 1852)	Doubtful species status.
Pteromalidae	*Pteromalusaurifacies* Förster, 1841	Doubtful species status.
Pteromalidae	*Pteromalusaurinitens* Förster, 1841	Doubtful species status.
Pteromalidae	*Pteromalusbarycerus* Förster, 1841	Doubtful species status.
Pteromalidae	*Pteromalusblandus* Förster, 1841	Doubtful species status.
Pteromalidae	*Pteromalusbreviscapus* Förster, 1841	Doubtful species status.
Pteromalidae	*Pteromalusbrunnicans* Ratzeburg, 1848	Doubtful species status.
Pteromalidae	*Pteromaluscarinatus* Förste,r 1841	Doubtful species status.
Pteromalidae	*Pteromaluscerinopus* Förster, 1841	Doubtful species status.
Pteromalidae	*Pteromaluschalcophanes* Förster, 1841	Doubtful species status.
Pteromalidae	*Pteromaluschalybaeus* Nees, 1834	Doubtful species status.
Pteromalidae	*Pteromaluschrysis* Förster, 1841	Doubtful species status.
Pteromalidae	*Pteromalusclavipes* Förster, 1841	Doubtful species status.
Pteromalidae	*Pteromaluscoerulescens* Ratzeburg, 1852	Doubtful species status.
Pteromalidae	*Pteromaluscoeruleus* Förster, 1841	Doubtful species status.
Pteromalidae	*Pteromaluscolosseus* Förster, 1841	Doubtful species status.
Pteromalidae	*Pteromaluscompactus* Förster, 1841	Doubtful species status.
Pteromalidae	*Pteromaluscompos* Förster, 1841	Doubtful species status.
Pteromalidae	*Pteromalusconcinnus* Förster, 1841	Doubtful species status.
Pteromalidae	*Pteromalusconoideus* Ratzeburg, 1848	Doubtful species status.
Pteromalidae	*Pteromaluscrassus* Förster, 1841	Doubtful species status.
Pteromalidae	*Pteromaluscryptocephali* Ratzeburg, 1852	Doubtful species status.
Pteromalidae	*Pteromaluscubocephalus* Förster ,1841	Doubtful species status.
Pteromalidae	*Pteromaluscupreus* Nees, 1834	Doubtful species status.
Pteromalidae	*Pteromaluscurculionoides* (Bouché, 1834)	Doubtful species status.
Pteromalidae	*Pteromaluscylindraceus* Förster, 1841	Doubtful species status.
Pteromalidae	*Pteromalusdahlbomi* Ratzeburg, 1844	Doubtful species status.
Pteromalidae	*Pteromalusdalmanni* Förster, 1841	Doubtful species status.
Pteromalidae	*Pteromalusdecipiens* Förster, 1841	Doubtful species status.
Pteromalidae	*Pteromalusdepressus* Förster, 1841	Doubtful species status.
Pteromalidae	*Pteromalusdevorator* Förster, 1841	Doubtful species status.
Pteromalidae	*Pteromalusdiadema* Ratzeburg, 1852	Doubtful species status.
Pteromalidae	*Pteromalusdiatatus* Schmidt, 1851	Doubtful species status.
Pteromalidae	*Pteromalusdifficilis* Förster, 1841	Doubtful species status.
Pteromalidae	*Pteromalusdimiduis* Dalla Torre, 1898	Doubtful species status.
Pteromalidae	*Pteromalusdiminuator* Förster ,1841	Doubtful species status.
Pteromalidae	*Pteromalusdirutor* Förster, 1841	Doubtful species status.
Pteromalidae	*Pteromalusdivitissimus* Dalla Torre, 1898	Doubtful species status.
Pteromalidae	*Pteromalusecarinatus* Förster, 1841	Doubtful species status.
Pteromalidae	*Pteromalusegregius* Förster, 1841	Doubtful species status.
Pteromalidae	*Pteromaluselatus* Förster, 1841	Doubtful species status.
Pteromalidae	*Pteromaluselongatus* Ratzeburg, 1852	Doubtful species status.
Pteromalidae	*Pteromaluseminens* Förster, 1841	Doubtful species status.
Pteromalidae	*Pteromalusesuriens* Förster, 1841	Doubtful species status.
Pteromalidae	*Pteromaluseuryops* Förster, 1861	Listed in Noyes (2019) for Gemany, but collecting location in Switzerland (Förster 1861).
Pteromalidae	*Pteromalusexiguus* Förster, 1841	Doubtful species status.
Pteromalidae	*Pteromalusexoletus* Förster, 1841	Doubtful species status.
Pteromalidae	*Pteromalusexsertus* Förster, 1841	Doubtful species status.
Pteromalidae	*Pteromalusextensus* Förster, 1841	Doubtful species status.
Pteromalidae	*Pteromalusfacilis* Förster, 1841	Doubtful species status.
Pteromalidae	*Pteromalusfagi* Ratzeburg, 1852	Doubtful species status.
Pteromalidae	*Pteromalusfaunigena* Förster, 1841	Doubtful species status.
Pteromalidae	*Pteromalusferox* Förster, 1841	Doubtful species status.
Pteromalidae	*Pteromalusfervidus* Förster, 1841	Doubtful species status.
Pteromalidae	*Pteromalusfestivus* Förster, 1841	Doubtful species status.
Pteromalidae	*Pteromalusflavipalpis* Ratzeburg, 1844	Doubtful species status.
Pteromalidae	*Pteromalusfugax* Förster, 1841	Doubtful species status.
Pteromalidae	*Pteromalusfurtivus* Förster, 1841	Doubtful species status.
Pteromalidae	*Pteromalusfuscopalpus* Förster, 1841	Doubtful species status.
Pteromalidae	*Pteromalusgenuinus* Förster, 1841	Doubtful species status.
Pteromalidae	*Pteromalusgermanicus* Özdikmen, 2011	Doubtful species status.
Pteromalidae	*Pteromalusgnavis* Förster, 1841	Doubtful species status.
Pteromalidae	*Pteromalusgracillimus* Dalla Torre, 1898	Doubtful species status.
Pteromalidae	*Pteromalusgratiosus* Förster, 1841	Doubtful species status.
Pteromalidae	*Pteromalusguttula* Ratzeburg, 1852	Doubtful species status.
Pteromalidae	*Pteromalushabilis* Förster, 1841	Doubtful species status.
Pteromalidae	*Pteromalusherbaceus* Förster, 1841	Doubtful species status.
Pteromalidae	*Pteromalushercyniae* Ratzeburg, 1844	Doubtful species status.
Pteromalidae	*Pteromalushonestus* Förster, 1841	Doubtful species status.
Pteromalidae	*Pteromalushyalopterus* Dalla Torre, 1898	Doubtful species status.
Pteromalidae	*Pteromalushypocyaneus* Förster, 1841	Doubtful species status.
Pteromalidae	*Pteromalusignobilis* Förster, 1861	Listed in Noyes (2019) for Gemany, but collecting location in Switzerland (Förster 1861).
Pteromalidae	*Pteromalusillustratus* Förster, 1841	Doubtful species status.
Pteromalidae	*Pteromalusimmundus* Förster, 1841	Doubtful species status.
Pteromalidae	*Pteromalusimpressifrons* Förster, 1841	Doubtful species status.
Pteromalidae	*Pteromalusinanis* Förster, 1841	Doubtful species status.
Pteromalidae	*Pteromalusincertus* Förster, 1841	Doubtful species status.
Pteromalidae	*Pteromalusinclytus* Förster, 1841	Doubtful species status.
Pteromalidae	*Pteromalusinconspicuus* Förster, 1841	Doubtful species status.
Pteromalidae	*Pteromalusinermis* Förster, 1841	Doubtful species status.
Pteromalidae	*Pteromalusinfelix* Dalla Torre, 1898	Doubtful species status.
Pteromalidae	*Pteromalusinfestus* Förster, 1841	Doubtful species status.
Pteromalidae	*Pteromalusinfinitus* Förster, 1841	Doubtful species status.
Pteromalidae	*Pteromalusinquilinus* Förster, 1841	Doubtful species status.
Pteromalidae	*Pteromalusinsignis* Förster, 1841	Doubtful species status.
Pteromalidae	*Pteromalusjejunus* Förster, 1841	Doubtful species status.
Pteromalidae	*Pteromaluslaetus* Förster, 1841	Doubtful species status.
Pteromalidae	*Pteromaluslaevis* Förster, 1842	Doubtful species status.
Pteromalidae	*Pteromaluslatreillei* Ratzeburg, 1848	Doubtful species status.
Pteromalidae	*Pteromaluslazulinus* Förster, 1841	Doubtful species status.
Pteromalidae	*Pteromaluslepidotus* Ratzeburg, 1852	Doubtful species status.
Pteromalidae	*Pteromalusleptogaster* Förster, 1841	Doubtful species status.
Pteromalidae	*Pteromalusleptostictus* Förster, 1841	Doubtful species status.
Pteromalidae	*Pteromaluslimbatus* Förster, 1841	Doubtful species status.
Pteromalidae	*Pteromaluslineolatus* Dalla Torre, 1898	Doubtful species status.
Pteromalidae	*Pteromaluslugens* Förster ,1841	Doubtful species status.
Pteromalidae	*Pteromaluslutulentus* Dalla Torre 1898	Doubtful species status.
Pteromalidae	*Pteromalusmacrocerus* Dalla Torre, 1898	Doubtful species status.
Pteromalidae	*Pteromalusmaculiscapus* Ratzeburg, 1844	Doubtful species status.
Pteromalidae	*Pteromalusmandibulatus* Dalla Torre, 1898	Doubtful species status.
Pteromalidae	*Pteromalusmariae* Dalla Torre, 1899	Doubtful species status.
Pteromalidae	*Pteromalusmaurus* Förster, 1841	Doubtful species status.
Pteromalidae	*Pteromalusmelancholicus* Förster, 1841	Doubtful species status.
Pteromalidae	*Pteromalusmelanocerus* Förster, 1841	Doubtful species status.
Pteromalidae	*Pteromalusmelanochlorus* Förster, 1841	Doubtful species status.
Pteromalidae	*Pteromalusmicros* Dalla Torre, 1898	Doubtful species status.
Pteromalidae	*Pteromalusmixtus* Förster, 1841	Doubtful species status.
Pteromalidae	*Pteromalusmobilis* Förster, 1841	Doubtful species status.
Pteromalidae	*Pteromalusmolestus* Förster, 1841	Doubtful species status.
Pteromalidae	*Pteromalusmonochrous* Förster, 1841	Doubtful species status.
Pteromalidae	*Pteromalusnanulus* Dalla Torre, 1898	Doubtful species status.
Pteromalidae	*Pteromalusnapaeus* Förster, 1841	Doubtful species status.
Pteromalidae	*Pteromalusnaucus* Förster, 1841	Doubtful species status.
Pteromalidae	*Pteromalusnavis* Ratzeburg, 1848	Doubtful species status.
Pteromalidae	*Pteromalusnebulosus* Dalla Torre, 1898	Doubtful species status.
Pteromalidae	*Pteromalusneesii* Ratzeburg, 1844	Doubtful species status.
Pteromalidae	*Pteromalusneglectus* Förster, 1841	Doubtful species status.
Pteromalidae	*Pteromalusnigricans* Förster, 1841	Doubtful species status.
Pteromalidae	*Pteromalusnobilis* Förster, 1841	Doubtful species status.
Pteromalidae	*Pteromalusnodulosus* Ratzeburg, 1848	Doubtful species status.
Pteromalidae	*Pteromalusnuperus* Förster, 1841	Doubtful species status.
Pteromalidae	*Pteromalusobductus* Förster, 1841	Doubtful species status.
Pteromalidae	*Pteromalusobscurus* Nees, 1834	Doubtful species status.
Pteromalidae	*Pteromalusobvolitans* Förster, 1841	Doubtful species status.
Pteromalidae	*Pteromalusopacus* Förster, 1841	Doubtful species status.
Pteromalidae	*Pteromalusopimus* Förster, 1841	Doubtful species status.
Pteromalidae	*Pteromalusornatus* Förster, 1841	Doubtful species status.
Pteromalidae	*Pteromaluspachygaster* Förster, 1841	Doubtful species status.
Pteromalidae	*Pteromaluspachymerus* Förster, 1841	Doubtful species status.
Pteromalidae	*Pteromaluspellucidiventris* Ratzeburg, 1848	Doubtful species status.
Pteromalidae	*Pteromaluspellucidus* Förster, 1841	Doubtful species status.
Pteromalidae	*Pteromaluspicinus* Förster, 1841	Doubtful species status.
Pteromalidae	*Pteromaluspilosellus* Förster, 1841	Doubtful species status.
Pteromalidae	*Pteromalusplaniusculus* Förster, 1841	Doubtful species status.
Pteromalidae	*Pteromaluspogonochoeri* Ratzeburg, 1844	Doubtful species status.
Pteromalidae	*Pteromaluspolychlori* Ratzeburg, 1852	Doubtful species status.
Pteromalidae	*Pteromaluspolycyclus* Förster, 1841	Doubtful species status.
Pteromalidae	*Pteromaluspomacearum* Ratzeburg, 1852	Doubtful species status.
Pteromalidae	*Pteromaluspraeceps* Förster, 1841	Doubtful species status.
Pteromalidae	*Pteromaluspraelongus* Förster, 1841	Doubtful species status.
Pteromalidae	*Pteromaluspraepes* Förster, 1841	Doubtful species status.
Pteromalidae	*Pteromaluspraepotens* Förster, 1841	Doubtful species status.
Pteromalidae	*Pteromalusprinceps* Förster, 1841	Doubtful species status.
Pteromalidae	*Pteromaluspropinquus* Förster, 1841	Doubtful species status.
Pteromalidae	*Pteromaluspsyllus* Förster, 1841	Doubtful species status.
Pteromalidae	*Pteromaluspulcherrimus* Förster, 1841	Doubtful species status.
Pteromalidae	*Pteromaluspullus* Förster, 1841	Doubtful species status.
Pteromalidae	*Pteromaluspunctum* Förster, 1841	Doubtful species status.
Pteromalidae	*Pteromaluspungens* Förster, 1841	Doubtful species status.
Pteromalidae	*Pteromaluspusillus* Förster, 1841	Doubtful species status.
Pteromalidae	*Pteromaluspygmaeanae* Ratzeburg, 1844	Doubtful species status.
Pteromalidae	*Pteromaluspygmaeus* Förster, 1841	Doubtful species status.
Pteromalidae	*Pteromalusquestionis* Förster, 1841	Doubtful species status.
Pteromalidae	*Pteromalusracemosi* Ratzeburg, 1848	Doubtful species status.
Pteromalidae	*Pteromalusramulorum* Ratzeburg, 1848	Doubtful species status.
Pteromalidae	*Pteromalusrapax* Förster, 1841	Doubtful species status.
Pteromalidae	*Pteromalusratzeburgii* Dalla Torre, 1898	Doubtful species status.
Pteromalidae	*Pteromalusregius* Förster, 1841	Doubtful species status.
Pteromalidae	*Pteromalusrelevatus* Förster, 1841	Doubtful species status.
Pteromalidae	*Pteromalusrhombicus* Förster, 1841	Doubtful species status.
Pteromalidae	*Pteromalussaltatorius* Förster, 1841	Doubtful species status.
Pteromalidae	*Pteromalussapphireus* Förster, 1841	Doubtful species status.
Pteromalidae	*Pteromalussimilis* Förster, 1841	Doubtful species status.
Pteromalidae	*Pteromalussimplex* Förster, 1841	Doubtful species status.
Pteromalidae	*Pteromalussincerus* Förster, 1841	Doubtful species status.
Pteromalidae	*Pteromalussingularis* Förster, 1841	Doubtful species status.
Pteromalidae	*Pteromalussmaragdinus* Förster, 1841	Doubtful species status.
Pteromalidae	*Pteromalussolidus* Förster, 1841	Doubtful species status.
Pteromalidae	*Pteromalussparsus* Förster, 1841	Doubtful species status.
Pteromalidae	*Pteromalussphaerogaster* Förster, 1841	Doubtful species status.
Pteromalidae	*Pteromalussplendidus* Förster, 1841	Doubtful species status.
Pteromalidae	*Pteromalusstrobilobius* Ratzeburg, 1852	Doubtful species status.
Pteromalidae	*Pteromalussubaequalis* Förster, 1841	Doubtful species status.
Pteromalidae	*Pteromalussublaevis* Förster, 1841	Doubtful species status.
Pteromalidae	*Pteromalussubpunctatus* Förster, 1841	Doubtful species status.
Pteromalidae	*Pteromalussubterraneus* Förster, 1841	Doubtful species status.
Pteromalidae	*Pteromalussulphuripes* Förster, 1841	Doubtful species status.
Pteromalidae	*Pteromalussybarita* Förster, 1841	Doubtful species status.
Pteromalidae	*Pteromalussylvarum* Förster, 1841	Doubtful species status.
Pteromalidae	*Pteromalussyntomus* Ratzeburg, 1852	Doubtful species status.
Pteromalidae	*Pteromalusterebrans* Förster, 1841	Doubtful species status.
Pteromalidae	*Pteromalustessellatus* Ratzeburg, 1852	Doubtful species status, in Vidal (2001) as *P.tesselatus* Ratzeburg.
Pteromalidae	*Pteromalustibialis* Nees, 1834	Doubtful species status.
Pteromalidae	*Pteromalustimidus* Dalla Torre, 1898	Doubtful species status.
Pteromalidae	*Pteromalustricollis* Förster, 1841	Doubtful species status.
Pteromalidae	*Pteromalustroglodytes* Dalla Torre, 1899	Doubtful species status.
Pteromalidae	*Pteromalusunicolor* Förster, 1841	Doubtful species status.
Pteromalidae	*Pteromalusuyari* Özdikmen, 2011	Doubtful species status, in Vidal (2001) as *P.aeneus* Nees.
Pteromalidae	*Pteromalusvaginatus* Förster, 1841	Doubtful species status.
Pteromalidae	*Pteromalusvaginulae* Ratzeburg, 1852	Doubtful species status.
Pteromalidae	*Pteromalusvallatus* Förster, 1841	Doubtful species status.
Pteromalidae	*Pteromalusvallecula* Ratzeburg, 1848	Doubtful species status.
Pteromalidae	*Pteromalusvariolosus* Förster, 1841	Doubtful species status.
Pteromalidae	*Pteromalusvelox* Förster, 1841	Doubtful species status.
Pteromalidae	*Pteromalusveneris* Dalla Torre, 1898	Doubtful species status.
Pteromalidae	*Pteromalusventricosus* Förster, 1841	Doubtful species status.
Pteromalidae	*Pteromalusverticalis* Förster, 1841	Doubtful species status.
Pteromalidae	*Pteromalusvicarius* Ratzeburg, 1852	Doubtful species status.
Pteromalidae	*Pteromalusvicinus* Förster, 1841	Doubtful species status.
Pteromalidae	*Pteromalusviolarum* Dalla Torre, 1898	Doubtful species status.
Pteromalidae	*Pteromalusviridicans* Förster, 1841	Doubtful species status, in Vidal (2001) as *P.viridicanus* Förster.
Pteromalidae	*Pteromalusvorax* Förster, 1841	Doubtful species status.
Pteromalidae	*Rhopalicusatricornis* (Förster, 1841)	Not included in Graham (1969).
Pteromalidae	*Rhopalicusmagdalis* (Ratzeburg ,1844)	Not included in Graham (1969).
Pteromalidae	*Rhopalicusopisthotomus* (Ratzeburg, 1848)	Not included in Graham (1969).
Pteromalidae	*Rhopalicusvirescens* (Ratzeburg 1844)	Not included in Graham (1969).
Pteromalidae	*Roptroceruspolychromus* Förster, 1856	Not included in Graham (1969).
Pteromalidae	*Roptrocerusxylobius* Förster, 1856	Not included in Graham (1969).
Pteromalidae	*Scutellistaobscura* (Förster, 1878)	Doubtful species status.
Pteromalidae	*Semiotelluspunctifrons* (Nees, 1834)	Doubtful species status.
Pteromalidae	*Sphegigasterclavicornis* Förster, 1841	Not included in Graham (1969), in Vidal (2001) as *Chrysolampusclavicornis* Förster.
Pteromalidae	*Stenomalinabicolor* Förster, 1841	Not included in Graham (1969).
Pteromalidae	*Stenomalinafallax* (Förster, 1841)	Not included in Graham (1969).
Pteromalidae	*Stenomalinaspectabilis* (Förster, 1841)	Graham (1969): possible synonym of *S.liparae* (Giraud).
Pteromalidae	*Trichomalopsispunctata* (Ratzeburg, 1844)	Not included in Graham (1969).
Pteromalidae	*Trichomaluscinctus* (Förster, 1841)	Not included in Graham (1969).
Pteromalidae	*Trichomaluscristatus* Förster, 1841	Graham (1969): ?Synonym of *T.posticus* (Walker).
Pteromalidae	*Trichomalusexquisitus* (Förster, 1841)	Not included in Graham (1969).
Pteromalidae	*Trichomalusfulgidus* (Förster, 1841)	Not included in Graham (1969).
Pteromalidae	*Trichomalusgeneralis* (Förster, 1841)	Not included in Graham (1969).
Pteromalidae	*Trichomalusglabellus* (Förster, 1841)	Not included in Graham (1969).
Pteromalidae	*Trichomalusintestinarius* (Förster, 1841)	Graham (1969): possible synonym of *T.posticus* (Walker).
Pteromalidae	*Trichomalusnotabilis* (Förster, 1841)	Not included in Graham (1969).
Pteromalidae	*Trichomalusobsessorius* (Förster, 1841)	Not included in Graham (1969).
Pteromalidae	*Trichomaluspilosus* (Ratzeburg, 1844)	Not included in Graham (1969).
Pteromalidae	*Trichomalopsiscaesareus* (Dalla Torre, 1889)	Nomen nudum according to Graham (1969) who writes: "Type ♀ (Germany) recognized by Delucchi (1958) who stated that it is a valid species. I have not seen the type and cannot recognize the species."
Pteromalidae	*Trigonoderusbimaculatus* (Nees, 1834)	Not included in Graham (1969).
Pteromalidae	*Trigonoderusimmaculatus* (Nees, 1834)	Not included in Graham (1969).
Pteromalidae	*Trigonoderusoccultus* (Förster, 1841)	Graham (1969): possible synonym of *T.filatus* Walker.
Torymidae	*Pseudotorymustarsatus* Nees, 1834	Graham and Gijswijt (1998): possible synonym of *P.sapphyrinus* (Fonscolombe).
Torymidae	*Torymusapproximatus* Förster, 1841	Doubtful species status.
Torymidae	*Torymusassociatus* Förster, 1841	Doubtful species status.
Torymidae	*Torymusconfluens* Ratzeburg, 1852	Doubtful species status.
Torymidae	*Torymusdifficilis* Nees, 1834	Doubtful species status.
Torymidae	*Torymuskaltenbachi* Förster, 1840	Doubtful species status.
Torymidae	*Torymusminutus* Förster, 1840	Doubtful species status.
Torymidae	*Torymusresinanae* Ratzeburg, 1852	Doubtful species status.
Trichogrammatidae	*Aphelinoideabischoffi* (Novicky, 1946)	Triapitsyn (2018): "Its listings from Germany (e.g. Viggiani (2011)) are incorrect, as both localities of the type series are in Burgenland, Austria, the other one (of the unspecified number of paratypes) being Jungerberg (near [W of] Jois, 47°57'38"N 16°46'44'E) between Jois and Winden am See (Novicky (1946): 46, as “Jungenberg”)."
